# Reductive Deuteration of Acyl Chlorides for the Synthesis of *α*,*α*-Dideuterio Alcohols Using SmI_2_ and D_2_O

**DOI:** 10.3390/molecules28010416

**Published:** 2023-01-03

**Authors:** Hengzhao Li, Yuxia Hou, Mengqi Peng, Lijun Wang, Junyu Li, Lei Ning, Zemin Lai, Yixuan Li, Jie An

**Affiliations:** 1 Department of Nutrition and Health, China Agricultural University, No. 2 Yuanmingyuan West Road, Beijing 100193, China; 2 Department of Chemistry and Innovation Center of Pesticide Research, China Agricultural University, No. 2 Yuanmingyuan West Road, Beijing 100193, China

**Keywords:** acyl chlorides, reductive deuteration, *α*,*α*-dideuterio alcohols, SmI_2_, D_2_O

## Abstract

The synthesis of *α*,*α*-dideuterio alcohols has been achieved via single electron transfer reductive deuteration of acyl chlorides using SmI_2_ and D_2_O. This method is distinguished by its remarkable functional group tolerance and exquisite deuterium incorporation, which has also been applied to the synthesis of valuable deuterated agrochemicals and their building blocks.

## 1. Introduction

Deuterated compounds have found wild applications in chemical analysis and drug discovery [1,2,3,4,5,6,7,8,9,10]. For example, deuterium-labeled internal standards are a major class of MS internal standards for the quantitative analysis of agrochemical residues in complex matrices [4,5,6,7]. Additionally, deuterated drugs have recently received much attention because of their improved pharmacokinetic profile and decreased toxicity [8,9,10].

**Scheme 1:**
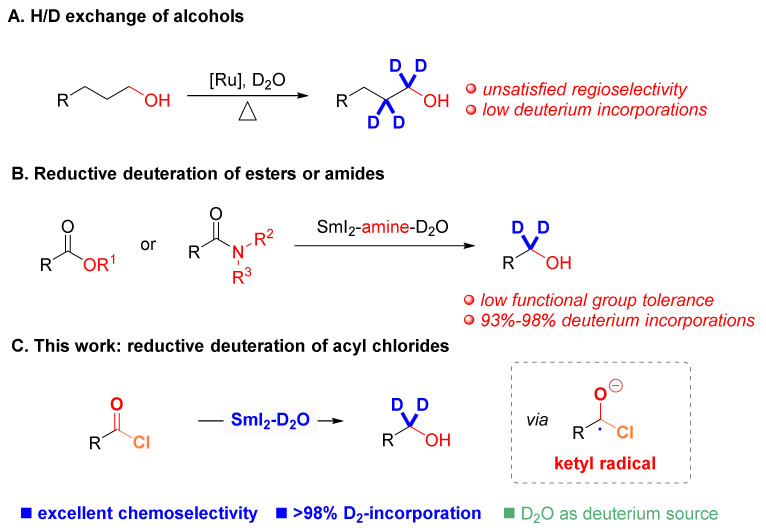
Synthesis of *α*,*α*-dideuterio alcohols.

Alcohols are among the most important and broadly utilized building blocks for the synthesis of agrochemicals and drugs [10,11,12,13,14,15,16]. Therefore, deuterium-labeled alcohols are valuable in the preparation of deuterated drugs and deuterated internal standards for agrochemical residue analysis. However, the synthesis of deuterated alcohols with high deuterium incorporations and chemoselectivity from readily available starting materials is still not straightforward [17,18,19,20,21,22,23,24]. Deuterated alcohols can be directly synthesized via the H/D exchange of alcohols (Scheme 1A) [18,19,20,21,22]. However, metal-catalyzed H/D exchange of alcohols often suffers from either unsatisfied regioselectivity or low deuterium incorporations [20,21,22]. The reductive deuteration of carboxylic acids and their derivatives is another strategy for the synthesis of deuterated alcohols [23,24,25]. Classic reductive deuteration requires the usage of expensive and pyrophoric LiAlD_4_ or NaBD_4_ [23,24]. In our previous work, SmI_2_-Et_3_N-D_2_O-mediated SET reductive deuteration reactions were developed to convert carboxylic acid derivatives into the corresponding deuterated alcohols (Scheme 1B) [25]. While the usage of Et_3_N as the ligand improved the reduction potential of SmI_2_, it decreased the chemoselectivity of those methods [25,26,27,28,29,30]. Et_3_N can only be avoided by using activated carboxylic acid derivatives, such as uncommercially available pentafluorophenyl esters and acyl fluorides [31,32]. In this study (Scheme 1C), we discovered that widely available acyl chlorides are more reactive ketyl precursors which are susceptible to SmI_2_-D_2_O. The reductive deuteration of acyl chloride is a four-electron transfer process. The ketyl radical shown in Scheme 1C was formed after the first electron transfer. The sequential electron transfers and deuterium cation transfers led to the formation of the deuterated alcohols. In the absence of an Et_3_N ligand, the reductive deuteration of acyl chlorides by SmI_2_-D_2_O gave excellent chemoselectivity and ≥98% deuterium incorporations.

## 2. Results

We began our investigation by examining the reductive deuteration of **1a** using SmI_2_ and D_2_O. The optimization studies demonstrated that the yield of **2a** was influenced by the amount of both SmI_2_ and D_2_O, while excellent deuterium incorporations were uniformly obtained under all the tested conditions. In this SET reductive deuteration reaction, D_2_O was both the deuterium donor and the ligand for SmI_2_ [31,32,33,34,35,36]. When 5 equiv. of SmI_2_ and 100 equiv. of D_2_O were used, 96% yield and 98% deuterium incorporation were obtained (Table 1, entry 1). Decreasing the amount of D_2_O from 100 equiv. to 75 equiv., the yield of **2a** was improved to 98% (Table 1, entry 2). The amount of D_2_O could be further decreased from 75 equiv. to 30 equiv. with the yields and deuterium incorporations maintained as the same (Table 1, entries 3−4). However, when the amount of D_2_O was less than 30 equiv., the yield of the reaction decreased with the decreasing of D_2_O (Table 1, entries 5−6). When 5 equiv. of SmI_2_ and 15 equiv. of D_2_O were used, only 80% of **2a** was formed with 20% carboxylic acid recovered (Table 1, entry 6). As the SET reduction of acyl chloride is a four-electron transfer process and SmI_2_ is a single electron donor, at least 4 equiv. of SmI_2_ is required. While the amount of SmI_2_ was decreased from 5 equiv. to 4 equiv., the yield was slightly decreased to 90%, which may be due to the side oxidation reaction between SmI_2_ and the oxygen (Table 1, entry 7). Finally, the effect of reaction time was studied. The results revealed that 5 min was enough to achieve full conversion and the half-life of this reaction was <30 s (Table 1, entries 8−9).

**Scheme 2:**
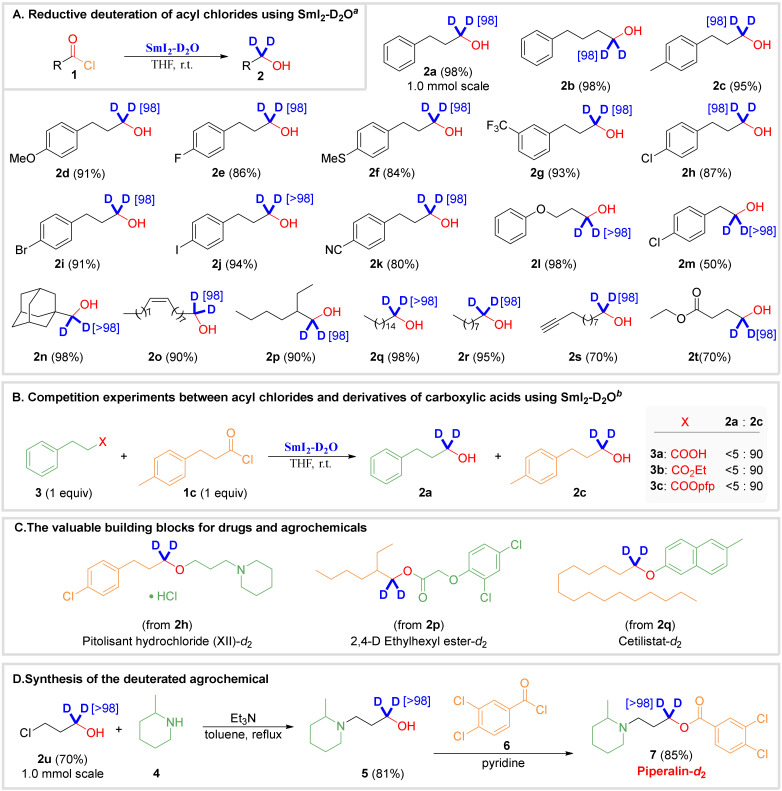
Reductive deuteration of acyl chlorides using SmI_2_ and D_2_O, competition studies and applications. *^a^* Conditions: D_2_O (30 equiv.) was added to the solution of SmI_2_ in THF (0.1 M, 5.0 equiv.), followed by **1** (0.30 mmol, 1.0 equiv.) in THF at r.t., and the resulting mixtures were stirred for 15 min under Ar. Isolated yield. *^b^* SmI_2_ (4.0 equiv.) and D_2_O (24 equiv.) were used. Yields and deuterium incorporations were determined by ^1^H NMR. pfp = C_6_F_5_.

With optimal conditions in hand, we next examined the generality of the reductive deuteration of acyl chlorides using SmI_2_ and D_2_O (Scheme 2). All the acyl chlorides surveyed were converted into the corresponding *α*,*α*-dideuterio alcohols with excellent deuterium incorporations and high yields (Scheme 2A). Functional groups such as chloride (**2h, 2m** and **2u**), bromide (**2i**), iodide (**2j**), alkyne (**2s**), methoxy (**2d**), thiomethyl (**2f**), alkenes (**2o**), and trifluoromethyl (**2g**) were all stable under the reaction conditions. Compared with the reductive deuteration protocols for carboxylic acids, esters, and amides using SmI_2_-Et_3_N-D_2_O, this method exhibited much better chemoselectivity. Acyl chloride could be reduced to *α*,*α*-dideuterio alcohols in 70% yield with full retention of the ethyl ester group (**2t**). Nitrile, another functional group that is sensitive to SmI_2_-Et_3_N-D_2_O, was also well tolerated with this method (**2k**). Next, a series of competition experiments between acyl chlorides (**1c**) and representative carboxylic acid derivatives (**3**) were conducted (Scheme 2B). Those studies revealed that the reduction of acyl chloride proceeded at a much higher rate compared with the reduction of carboxylic acid (**3a**) and the ester (**3b** and **3c**). Of note, although activated pentafluorophenyl esters can be reduced by SmI_2_-D_2_O as well, excellent selectivity with pentafluorophenyl esters (**3c**) was still obtained, which highlighted the remarkable chemoselectivity of this reaction and exhibited that acyl chlorides were the most reactive ketyl precursor under SmI_2_-mediated conditions by far. Finally, 1 mmol scale reactions were conducted which also resulted in ≥98% D_2_ incorporations (**2a** and **2u**).

This method has been applied in the synthesis of valuable deuterium-labeled building blocks for deuterated agrochemicals and drugs (Scheme 2C). By using the *α*,*α*-dideuterio alcohol building blocks (**2h**, **2p**, **2q**, and **2u**) synthesized in this work, the deuterium labeled pitolisant hydrochloride (XII), 2,4-D ethylhexyl ester, cetilistat, and piperalin can be readily prepared via known protocols [14,15,37,38]. None of these deuterated bioactive molecules have been investigated in the literature to date, which potentially could be used as deuterated drug candidates or MS internal standards.

Among them, piperalin is a widely used pesticide for the control of powdery mildews on ornamentals [38]. Deuterium-labeled piperalin will be useful as an internal standard for the accurate analysis of its residue in complex matrices. Therefore, we accomplished the synthesis of deuterated piperalin **7**. In this synthesis (Scheme 2D), *α*,*α*-dideuterio alcohol **2u** was first synthesized by the reductive deuteration of **1u** using SmI_2_–D_2_O. Next, nucleophilic attack on chloride **2u** by amine **4** led to the formation of compound **5**. Finally, **5** was treated with acyl chloride **6** to give piperalin-*d*_2_ **7** in 85% yield. Of note, >98% D_2_ incorporation was maintained in the final product **7**. This example showed the potential of this protocol in the synthesis of valuable deuterated molecules.

## 3. Materials and Methods

### 3.1. General Information

The glassware used in this study was dried in an oven overnight. Thin-layer chromatography analysis was carried out on SIL G/UV254 silica-glass plates. The plates were visualized using a 254 nm ultraviolet lamp or KMnO_4_ solution. All flash column chromatography was performed using silica gel 60, 35−70 μm. Additionally, ^1^H and ^13^C{^1^H} NMR spectra were recorded in CDCl_3_ on a Bruker spectrometer at 300 or 500 MHz. Data were manipulated directly from the spectrometer or via a networked personal computer with the appropriate software. All shifts are relative to the residual CHCl_3_ peak (7.27 for ^1^H NMR and 77.1 for ^13^C{^1^H} NMR). Multiplicities for coupled signals were designated using the following abbreviations: s, singlet; d, doublet; t, triplet; q, quartet; and br, broad signal. All coupling constants (J) are reported in hertz (Hz).

All solvents and reagents used in this study were purchased from commercial sources and used without further purification. SmI_2_ (0.1 M in THF) [39] and acyl chlorides [40] were synthesized by previously reported methods. D_2_O with 99.9% deuterium incorporation was used in this study. All the products obtained in this study are known compounds. The Supplementary Materials provide the ^1^H and ^13^C{^1^H} NMR spectra of **2a**–**2u, 5** and **7**.

Deuterium incorporation was determined by ^1^H NMR according to the equation below:

Deuterium incorporation = 100% − (area R-CH_2_-OH/2)

area R-CH_2_-OH means the integration of the R-CH_2_-OH peak.

### 3.2. Optimization Studies (Table 1)

To a solution of SmI_2_ (0.1 M in THF; 12.0−15.0 mL, 1.20−1.50 mmol, 4.00 equiv.−5.00 equiv.) and D_2_O (90.0 mg−600 mg, 4.50 mmol−30.0 mmol, 15.0 equiv.−100 equiv.), the solution of **1a** (50.6 mg, 0.300 mmol, 1.00 equiv.) in THF (1.0 mL) was added under Ar. The reaction mixture was stirred vigorously at r.t. for 0.50−15 min. Then, excess SmI_2_ was quenched by bubbling air into the reaction mixture. The reaction mixture was then diluted with EtOAc (10 mL) and HCl (5 mL, 1.0 M, aq). The aqueous layer was extracted with EtOAc (3 × 10 mL). The combined organic layers were washed with Na_2_S_2_O_3_ (5 mL, sat., aq). The organic layers were then dried over Na_2_SO_4_, filtered, and concentrated. The deuterium incorporation and the yield of the sample were obtained by ^1^H NMR (CDCl_3_, 300 MHz) using 1,1,2,2-tetrachloroethane as the internal standard.

### 3.3. General Procedure for the Reductive Deuteration of Acyl Chlorides by SmI_2_-D_2_O (Scheme 2A)

To a solution of SmI_2_ (0.1 M in THF; 15.0 mL, 1.50 mmol, 5.00 equiv.) and D_2_O (180 mg, 9.00 mmol, 30.0 equiv.), the solution of acyl chlorides (0.300 mmol, 1.00 equiv.) in THF (1.0 mL) was added under Ar. The reaction mixture was stirred vigorously at r.t. for 15 min. Then, excess SmI_2_ was quenched by bubbling air into the reaction mixture. The reaction mixture was diluted with EtOAc (10 mL) and HCl (5 mL, 1.0 M, aq). The aqueous layer was extracted with EtOAc (3 × 10 mL), and organic layers were combined and washed with Na_2_S_2_O_3_ (5 mL, sat., aq). The organic layers were then dried over Na_2_SO_4_, filtered, and concentrated. The crude product was purified by flash chromatography (silica, 0–25% hexane/EtOAc).

3-Phenylpropan-1,1-*d*_2_-1-ol (**2a**) [33]: colorless oil (135 mg, 98%, 1.00 mmol) (D_2_ incorporation 98%). ^1^H NMR (300 MHz, CDCl_3_) δ 7.34–7.28 (m, 2H), 7.25–7.17 (m, 3H), 2.73 (t, *J* = 7.7 Hz, 2H), 1.90 (t, *J* = 7.7 Hz, 2H), 1.44 (br, 1H); ^13^C{^1^H} NMR (75 MHz, CDCl_3_) δ 142.5, 128.5 (×2), 125.9, 61.6 (m), 34.1, 32.1.

4-Phenylbutan-1,1-*d*_2_-1-ol (**2b**) [41]: colorless oil (44.8 mg, 98%) (D_2_ incorporation 98%). ^1^H NMR (300 MHz, CDCl_3_) δ 7.32–7.24 (m, 2H), 7.22–7.14 (m, 3H), 2.64 (t, *J* = 7.5 Hz, 2H), 1.69 (m, 2H), 1.59 (m, 2H); ^13^C{^1^H} NMR (75 MHz, CDCl_3_) δ 142.4, 128.5, 128.4, 125.8, 62.1 (m), 35.7, 32.2, 28.1.

3-(*p*-Tolyl)propan-1,1-*d*_2_-1-ol (**2c**) [33]: colorless oil (43.4 mg, 95%) (D_2_ incorporation 98%). ^1^H NMR (300 MHz, CDCl_3_) δ 7.14–7.10 (m, 4H), 2.69 (t, *J* = 7.7 Hz, 2H), 2.34 (s, 3H), 1.88 (t, *J* = 7.7 Hz, 2H), 1.64 (br, 1H); ^13^C{^1^H} NMR (75 MHz, CDCl_3_) δ 138.8, 135.4, 129.2, 128.4, 61.6 (m), 34.2, 31.7, 21.0.

3-(4-Methoxyphenyl)propan-1,1-*d*_2_-1-ol (**2d**) [33]: colorless oil (45.9 mg, 91%) (D_2_ incorporation 98%). ^1^H NMR (300 MHz, CDCl_3_) δ 7.13 (m, 2H), 6.85 (m, 2H), 3.80 (s, 3H), 2.66 (t, *J* = 7.6 Hz, 2H), 1.86 (t, *J* = 7.6 Hz, 2H), 1.52 (br, 1H); ^13^C{^1^H} NMR (75 MHz, CDCl_3_) δ 157.9, 134.0, 129.4, 113.9, 61.6 (m), 55.3, 34.3, 31.2.

3-(4-Fluorophenyl)propan-1,1-*d*_2_-1-ol (**2e**) [33]: colorless oil (40.3 mg, 86%) (D_2_ incorporation 98%). ^1^H NMR (300 MHz, CDCl_3_) δ 7.17 (m, 2H), 6.98 (m, 2H), 2.69 (t, *J* = 7.7 Hz, 2H), 1.86 (t, *J* = 7.7 Hz, 2H), 1.31 (br, 1H); ^13^C{^1^H} NMR (75 MHz, CDCl_3_) δ 161.4 (d, *J*_C-F_ = 243.4 Hz), 137.5 (d, *J*_C-F_ = 3.2 Hz), 129.8 (d, *J*_C-F_ = 7.7 Hz), 115.2 (d, *J*_C-F_ = 21.1 Hz), 61.4 (m), 34.2, 31.3.

3-(4-(Methylthio)phenyl)propan-1,1-*d*_2_-1-ol (**2f**) [33]: colorless oil (46.4 mg, 84%) (D_2_ incorporation 98%). ^1^H NMR (300 MHz, CDCl_3_) δ 7.22 (m, 2H), 7.14 (m, 2H), 2.68 (t, *J* = 7.7 Hz, 2H), 2.48 (s, 3H), 1.87 (t, *J* = 7.7 Hz, 2H), 1.26 (br, 1H); ^13^C{^1^H} NMR (75 MHz, CDCl_3_) δ 139.1, 135.5, 129.1, 127.4, 61.5 (m), 34.0, 31.5, 17.2.

3-(3-(Trifluoromethyl)phenyl)propan-1,1-*d*_2_-1-ol (**2g**) [33]: colorless oil (57.5 mg, 93%) (D_2_ incorporation 98%). ^1^H NMR (300 MHz, CDCl_3_) δ 7.51–7.44 (m, 2H), 7.43–7.36 (m, 2H), 2.79 (t, *J* = 7.8 Hz, 2H), 1.90 (t, *J* = 7.8 Hz, 2H), 1.45 (br, 1H); ^13^C{^1^H} NMR (75 MHz, CDCl_3_) δ 142.8, 131.9, 130.8 (q, *J*_C-F_ = 31.8 Hz), 128.9, 125.2 (q, *J*_C-F_ = 3.8 Hz), 124.3 (q, *J*_C-F_ = 273.1 Hz), 122.9 (q, *J*_C-F_ = 3.9 Hz), 61.3 (m), 33.8, 31.9.

3-(4-Chlorophenyl)propan-1,1-*d*_2_-1-ol (**2h**) [33]: colorless oil (45.1 mg, 87%) (D_2_ incorporation 98%). ^1^H NMR (300 MHz, CDCl_3_) δ 7.25 (m, 2H), 7.12 (m, 2H), 2.68 (t, *J* = 7.7 Hz, 2H), 1.85 (t, *J* = 7.7 Hz, 2H), 1.46 (br, 1H); ^13^C{^1^H} NMR (75 MHz, CDCl_3_) δ 140.3, 131.7, 129.8, 128.6, 61.3 (m), 33.9, 31.4.

3-(4-Bromophenyl)propan-1,1-*d*_2_-1-ol (**2i**) [33]: colorless oil (59.3 mg, 91%) (D_2_ incorporation 98%). ^1^H NMR (300 MHz, CDCl_3_) δ 7.41 (m, 2H), 7.08 (m, 2H), 2.67 (t, *J* = 7.7 Hz, 2H), 1.85 (t, *J* = 7.7 Hz, 2H), 1.38 (br, 1H); ^13^C{^1^H} NMR (75 MHz, CDCl_3_) δ 140.9, 131.5, 130.3, 119.7, 61.3 (m), 33.9, 31.5.

3-(4-Iodophenyl)propan-1,1-*d*_2_-1-ol (**2j**) [33]: colorless oil (74.5 mg, 94%) (D_2_ incorporation > 98%). ^1^H NMR (300 MHz, CDCl_3_) δ 7.61 (m, 2H), 6.96 (m, 2H), 2.66 (t, *J* = 7.7 Hz, 2H), 1.85 (t, *J* = 7.7 Hz, 2H), 1.40 (br, 1H); ^13^C{^1^H} NMR (75 MHz, CDCl_3_) δ 141.5, 137.5, 130.6, 90.9, 61.3 (m), 33.8, 31.6.

4-(3-Hydroxypropyl-3,3-*d*_2_)benzonitrile (**2k**) [33]: white solid (39.2 mg, 80%) (D_2_ incorporation 98%). ^1^H NMR (300 MHz, CDCl_3_) δ 7.57 (m, 2H), 7.31 (m, 2H), 2.78 (t, *J* = 7.8 Hz, 2H), 1.88 (t, *J* = 7.8 Hz, 2H), 1.57 (br, 1H); ^13^C{^1^H} NMR (75 MHz, CDCl_3_) δ 147.7, 132.3, 129.3, 119.1, 109.8, 61.0 (m), 33.4, 32.2.

3-Phenoxypropan-1,1-*d*_2_-1-ol (**2l**) [42]: colorless oil (45.3 mg, 98%) (D_2_ incorporation > 98%). ^1^H NMR (300 MHz, CDCl_3_) δ 7.33–7.23 (m, 2H), 7.00–6.86 (m, 3H), 4.12 (t, *J* = 5.9 Hz, 2H), 2.03 (t, *J* = 5.9 Hz, 2H), 1.91 (br, 1H); ^13^C{^1^H} NMR (75 MHz, CDCl_3_) δ 158.8, 129.6, 121.0, 114.6, 65.7, 59.9 (m), 31.9.

2-(4-Chlorophenyl)ethan-1,1-*d*_2_-1-ol (**2m**) [43]: colorless oil (23.8 mg, 50%) (D_2_ incorporation > 98%). ^1^H NMR (300 MHz, CDCl_3_) δ 7.29 (m, 2H), 7.18 (m, 2H), 2.84 (s, 2H), 1.37 (br, 1H); ^13^C{^1^H} NMR (75 MHz, CDCl_3_) δ 137.1, 132.4, 130.4, 128.8, 62.8 (m), 38.4.

((3*r*,5*r*,7*r*)-Adamantan-1-yl)methan-*d*_2_-ol (**2n**) [33]: white solid (49.5 mg, 98%) (D_2_ incorporation > 98%). ^1^H NMR (300 MHz, CDCl_3_) δ 1.99 (m, 3H), 1.69 (m, 6H), 1.51 (m, 6H), 1.43 (br, 1H); ^13^C{^1^H} NMR (75 MHz, CDCl_3_) δ 73.1 (m), 39.1, 37.3, 34.4, 28.3.

(*Z*)-Octadec-9-en-1,1-*d*_2_-1-ol (**2o**) [33]: colorless oil (73.0 mg, 90%) (D_2_ incorporation 98%). ^1^H NMR (300 MHz, CDCl_3_) δ 5.35 (m, 2H), 2.02 (m, 4H), 1.55 (m, 2H), 1.40–1.24 (m, 22H), 0.88 (m, 3H); ^13^C{^1^H} NMR (75 MHz, CDCl_3_) δ 130.0, 129.9, 62.4 (m), 32.7, 32.0, 29.9, 29.8, 29.6 (×2), 29.5, 29.4 (×2), 29.3, 27.3 (×2), 25.8, 22.8, 14.2.

2-Ethylhexan-1,1-*d*_2_-1-ol (**2p**) [33]: colorless oil (35.7 mg, 90%) (D_2_ incorporation 98%). ^1^H NMR (300 MHz, CDCl_3_) δ 1.43–1.25 (m, 9H), 0.98–0.82 (m, 6H); ^13^C{^1^H} NMR (75 MHz, CDCl_3_) δ 64.7 (m), 41.9, 30.2, 29.2, 23.4, 23.2, 14.2, 11.2.

Hexadecan-1,1-*d*_2_-1-ol (**2q**) [33]: colorless oil (71.9 mg, 98%) (D_2_ incorporation > 98%). ^1^H NMR (300 MHz, CDCl_3_) δ 1.55 (m, 2H), 1.37–1.21 (m, 26H), 0.88 (t, *J* = 6.6 Hz, 3H); ^13^C{^1^H} NMR (75 MHz, CDCl_3_) δ 62.4 (m), 32.7, 32.0, 29.8 (×4), 29.7 (×4), 29.5, 29.4, 25.8, 22.8, 14.2.

Nonan-1,1-*d*_2_-1-ol (**2r**) [44]: colorless oil (41.7 mg, 95%) (D_2_ incorporation 98%). ^1^H NMR (300 MHz, CDCl_3_) δ 1.75 (br, 1H), 1.55 (m, 2H), 1.40–1.22 (m, 12H), 0.88 (t, *J* = 7.0 Hz, 3H); ^13^C{^1^H} NMR (75 MHz, CDCl_3_) δ 62.4 (m), 32.7, 32.0, 29.6, 29.5, 29.3, 25.8, 22.7, 14.1.

Undec-10-yn-1,1-*d*_2_-1-ol (**2s**) [33]: colorless oil (35.8 mg, 70%) (D_2_ incorporation 98%). ^1^H NMR (300 MHz, CDCl_3_) δ 2.17 (td, *J* = 7.1, 2.6 Hz, 2H), 1.94 (t, *J* = 2.6 Hz, 1H), 1.60–1.47 (m, 4H), 1.43–1.26 (m, 10H); ^13^C{^1^H} NMR (75 MHz, CDCl_3_) δ 84.8, 68.1, 62.4 (m), 32.7, 29.5, 29.4, 29.1, 28.8, 28.6, 25.7, 18.5.

Ethyl 4-hydroxybutanoate-4,4-*d*_2_ (**2t**) [31]: colorless oil (28.2 mg, 70%) (D_2_ incorporation 98%). ^1^H NMR (300 MHz, CDCl_3_) δ 4.14 (q, *J* = 7.2 Hz, 2H), 2.44 (t, *J* = 7.1 Hz, 2H), 1.88 (t, *J* = 7.2 Hz, 2H), 1.26 (t, *J* = 7.1 Hz, 3H); ^13^C{^1^H} NMR (75 MHz, CDCl_3_) δ 174.0, 61.5 (m), 60.6, 31.1, 27.6, 14.3.

3-Chloropropan-1,1-*d*_2_-1-ol (**2u**) [45]: colorless oil (67.6 mg, 70%, 1.00 mmol) (D_2_ incorporation > 98%). ^1^H NMR (500 MHz, CDCl_3_) δ 3.69 (t, *J* = 6.3 Hz, 2H), 2.00 (t, *J* = 6.3 Hz, 2H), 1.82 (br, 1H); ^13^C{^1^H} NMR (126 MHz, CDCl_3_) δ 58.7 (m), 41.7, 34.7.

### 3.4. Competition Experiments (Scheme 2B)

To a solution of SmI_2_ (0.1 M in THF; 8.00 mL, 0.800 mmol, 4.00 equiv.) and D_2_O (96.0 mg, 4.80 mmol, 24.0 equiv.), the solution of **3** (0.200 mmol, 1.00 equiv.) and **1c** (0.200 mmol, 1.00 equiv.) in THF (1.0 mL) was added under Ar. The reaction mixture was stirred vigorously at r.t. for 15 min. Then, excess SmI_2_ was quenched by bubbling air into the reaction mixture. The reaction mixture was then diluted with EtOAc (10 mL) and HCl (5 mL, 1.0 M, aq). The aqueous layer was extracted with EtOAc (3 × 10 mL), and organic layers were combined and washed with Na_2_S_2_O_3_ (5 mL, sat., aq). The organic layers were then dried over Na_2_SO_4_, filtered, and concentrated. The relative quantity of each product was obtained by ^1^H NMR (CDCl_3_, 300 MHz) using 1,1,2,2-tetrachloroethane as the internal standard.

### 3.5. Synthesis of Piperalin-d_2_ (Scheme 2D)

To a solution of **2u** (48.3 mg, 0.500 mmol, 1.00 equiv.) in toluene (2.00 mL), 2-methylpiperidine **4** (59.5 mg, 0.600 mmol, 1.20 equiv.) and Et_3_N (60.7 mg, 0.600 mmol, 1.20 equiv.) were added at r.t. The reaction mixture was then warmed to 110 ℃ and stirred vigorously overnight. Next, the reaction mixture was cooled to r.t. and diluted with EtOAc (10.0 mL) and NaOH (5.00 mL, 1.00 M, aq). The aqueous layer was extracted with EtOAc (3 × 10.0 mL). Organic layers were combined, dried over Na_2_SO_4_, filtered, and concentrated. The crude product was purified by flash chromatography (silica, 11–25% hexane/EtOAc), afforded 64.5 mg of 3-(2-methylpiperidin-1-yl)propan-1,1-*d*_2_-1-ol **5** in 81% yield as a yellow oil. D_2_ incorporation was >98%. ^1^H NMR (500 MHz, CDCl_3_) δ 2.96 (m, 2H), 2.33 (m, 2H), 2.05 (m, 1H), 1.79 (m, 1H), 1.66–1.43 (m, 5H), 1.27 (m, 2H), 1.08 (d, *J* = 6.3 Hz, 3H); ^13^C{^1^H} NMR (126 MHz, CDCl_3_) δ 63.9 (m), 56.6, 54.2, 51.5, 34.6, 27.2, 25.9, 23.2, 18.2.

To a solution of **5** (47.8 mg, 0.300 mmol, 1.00 equiv.) and pyridine (28.5 mg, 0.360 mmol, 1.20 equiv.) in CH_2_Cl_2_ (2.00 mL), 3,4-dichlorobenzoyl chloride **6** (75.4 mg, 0.360 mmol, 1.20 equiv.) was added at 0 ℃. The reaction mixture was stirred vigorously overnight at r.t. Then, the reaction mixture was diluted with EtOAc (10.0 mL) and NaOH (5.00 mL, 1.00 M, aq). The aqueous layer was extracted with EtOAc (3 × 10.0 mL). Organic layers were combined, dried over Na_2_SO_4_, filtered, and concentrated. The crude product was purified by flash chromatography (silica, 11–25% hexane/EtOAc), afforded 84.7 mg of piperalin-*d*_2_ **7** in 85% yield as a yellow oil. (D_2_ incorporation > 98%). ^1^H NMR (500 MHz, CDCl_3_) δ 8.08 (m, 1H), 7.83 (m, 1H), 7.50 (m, 1H), 2.83 (m, 2H), 2.43 (m, 1H), 2.26 (m, 1H), 2.13 (m, 1H), 1.90 (t, *J* = 7.5 Hz, 2H), 1.70–1.47 (m, 4H), 1.28 (m, 2H), 1.05 (d, *J* = 6.3 Hz, 3H); ^13^C{^1^H} NMR (126 MHz, CDCl_3_) δ 164.7, 137.5, 132.9, 131.5, 130.5, 130.3, 128.6, 63.8 (m), 55.9, 52.2, 50.3, 34.6, 26.2, 24.9, 23.9, 19.1.

## 4. Conclusions

In summary, widely available acyl chlorides were discovered as the most reactive ketyl precursors that were susceptible to mild SmI_2_-D_2_O. The reductive deuteration reaction protocol of acyl chloride has been developed for the synthesis of *α*,*α*-dideuterio alcohols. This new protocol features remarkable functional group tolerance and exquisite levels of D_2_ incorporations. The synthetic utility of this strategy was showcased in the preparation of deuterated piperalin and a series of deuterated building blocks. Further applications of this method in the synthesis of valuable deuterated agrochemicals are ongoing and will be reported in due course.

## Figures and Tables

**Table 1:** Optimization studies of the reductive deuteration of acyl chlorides using SmI_2_-D_2_O *^a^*.

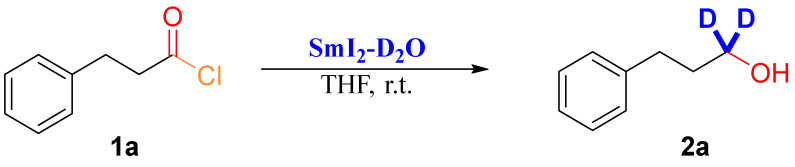					
Entry	SmI_2_ (equiv.)	D_2_O (equiv.)	Time (min)	Yield *^b^* (%)	[D_2_] *^b^* (%)
1	5	100	15	96	98
2	5	75	15	>98	98
3	5	60	15	>98	97
4	5	30	15	>98	98
5	5	25	15	95	97
6	5	15	15	80	97
7	4	30	15	90	97
8	5	30	5	95	97
9	5	30	0.5	78	98

*^a^* Conditions: D_2_O was added to a solution of SmI_2_ in THF, followed by **1a** in THF at r.t., and the resulting mixtures were stirred under Ar. *^b^* Determined using ^1^H NMR.

## Data Availability

All data obtained are contained in this article.

## Supplementary Materials

The following supporting information can be downloaded at: https://www.mdpi.com/article/10.3390/molecules28010416/s1, ^1^H and ^13^C{^1^H} NMR spectra for all compounds.

## Sample Availability

Samples of the compounds are not available from the authors.
