# Supplementary material for: Reductive Deuteration of Acyl Chlorides for the Synthesis of α,α-Dideuterio Alcohols Using SmI2 and D2O
Source: Molecules. 2023 Jan 3;28(1):416. doi: 10.3390/molecules28010416 (PMC9823311; doi:10.3390/molecules28010416)
Supplement: Supplementary file 1 [file molecules-28-00416-s001.zip › molecules-2099054-supplementary.pdf]

Reductive Deuteration of Acyl Chlorides for the Synthesis of α, α-Dideuterio Alcohols Using SmI_2 and D_2O

Hengzhao Li [1, 2], Yuxia Hou [2], Mengqi Peng [1, 2], Lijun Wang [1, 2], Junyu Li [1], Lei Ning [1], Zemin Lai [1], Yixuan Li [1, *] and Jie An [1, *]

[1]Department of Nutrition and Health, China Agricultural University, Beijing 100193, China

[2]College of Science, China Agricultural University, Beijing 100193, China

Supplementary Information

^{1}H and ^{13}C{^{1}H} NMR Spectra of Products

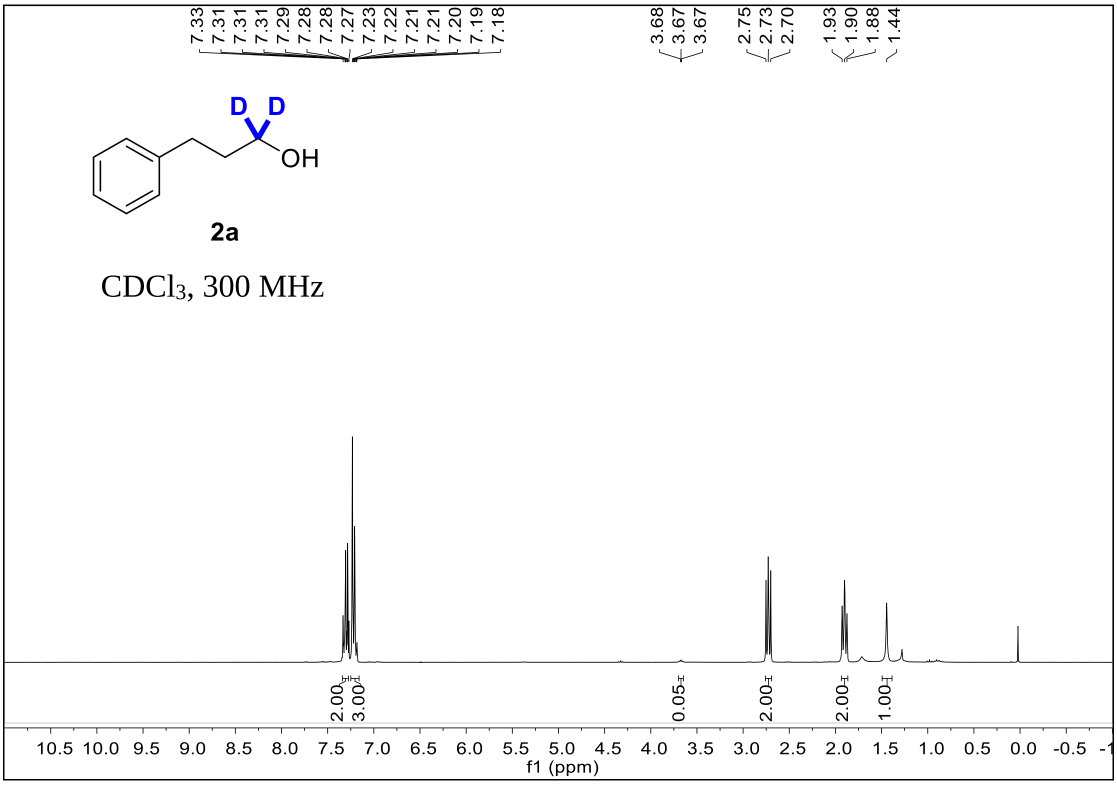
D D
OH
2a
CDCl3, 300 MHz
7.33 7.31 7.31 7.31 7.29 7.28 7.28 7.27 7.23 7.22 7.21 7.21 7.20 7.19 7.18
3.68 3.67 3.67
2.75 2.73 2.70
1.93 1.90 1.88
1.44
2.00 3.00 0.05 2.00 2.00 1.00
10.5 10.0 9.5 9.0 8.5 8.0 7.5 7.0 6.5 6.0 5.5 5.0 4.5 4.0 3.5 3.0 2.5 2.0 1.5 1.0 0.5 0.0 -0.5
f1 (ppm)

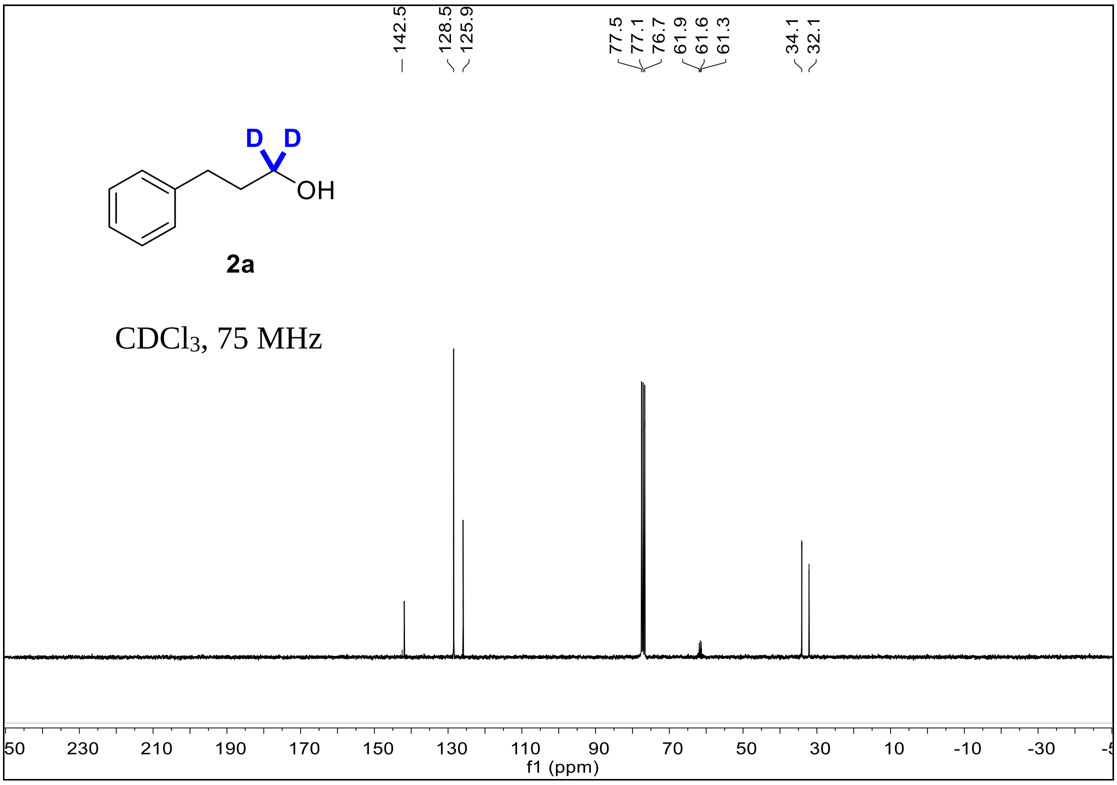
D D
OH
2a
CDCl3, 75 MHz
142.5
128.5 125.9
77.5 77.1 76.7
61.9 61.6 61.3
34.1 32.1
230 210 190 170 150 130 110 90 70 50 30 10 -10 -30
f1 (ppm)

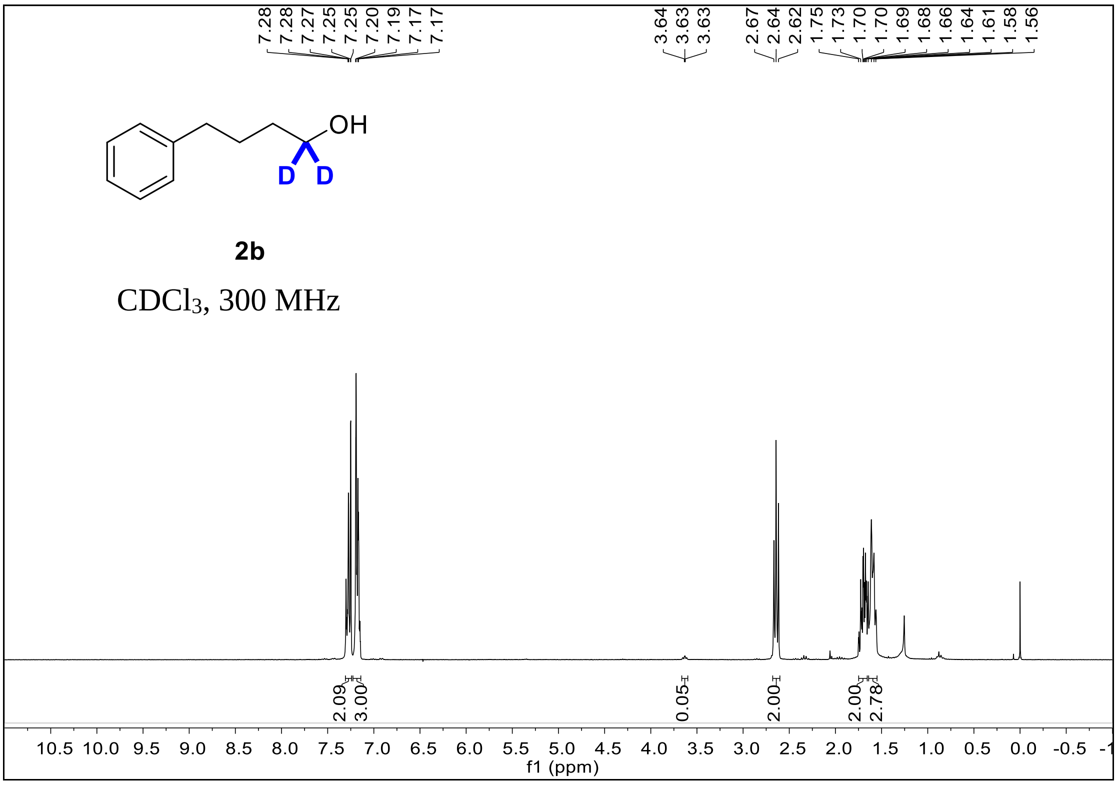
7.28 7.28 7.27 7.25 7.25 7.20 7.19 7.17 7.17
3.64 3.63 3.63
2.67 2.64 2.62 1.75 1.73 1.70 1.70 1.69 1.68 1.66 1.64 1.61 1.58 1.56
OH
D D
2b
CDCl3, 300 MHz
2.09 3.00 0.05 2.00 2.00 2.78
10.5 10.0 9.5 9.0 8.5 8.0 7.5 7.0 6.5 6.0 5.5 5.0 4.5 4.0 3.5 3.0 2.5 2.0 1.5 1.0 0.5 0.0 -0.5
f1 (ppm)

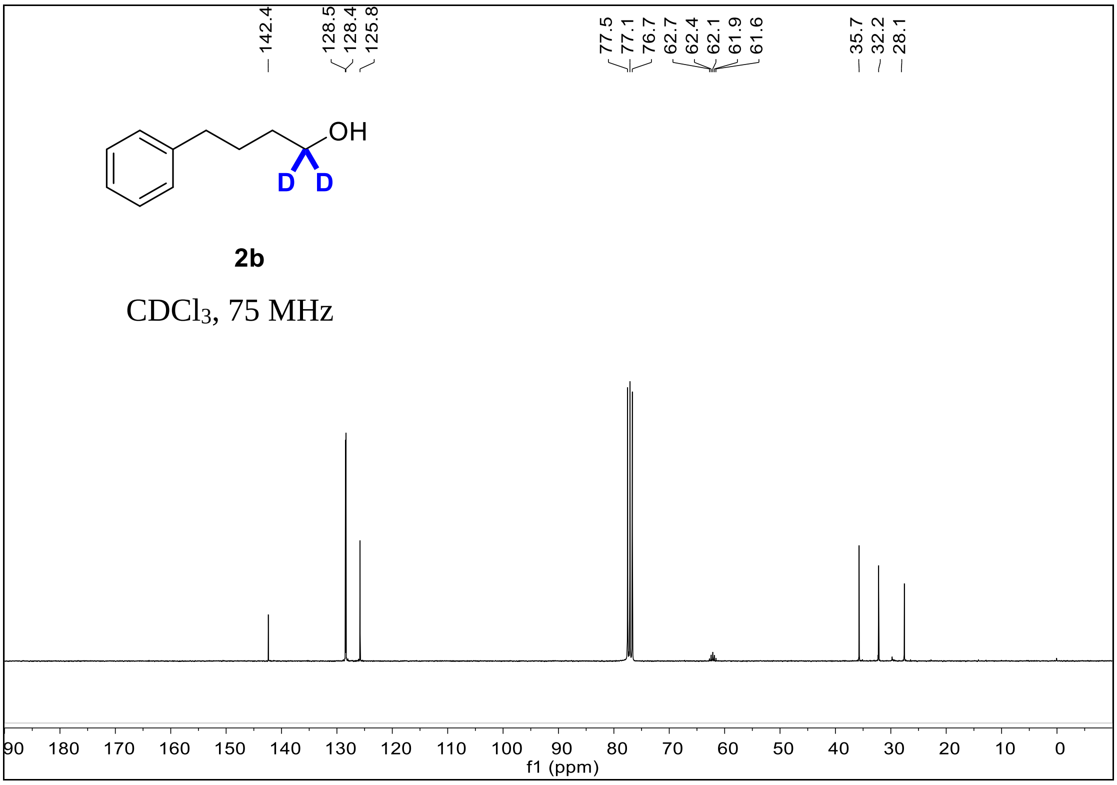
142.4
128.5 128.4 125.8
77.5 77.1 76.7 62.7 62.4 62.1 61.9 61.6
35.7 32.2 28.1
OH
D D
2b
CDCl3, 75 MHz
180 170 160 150 140 130 120 110 100 90 80 70 60 50 40 30 20 10 0
f1 (ppm)

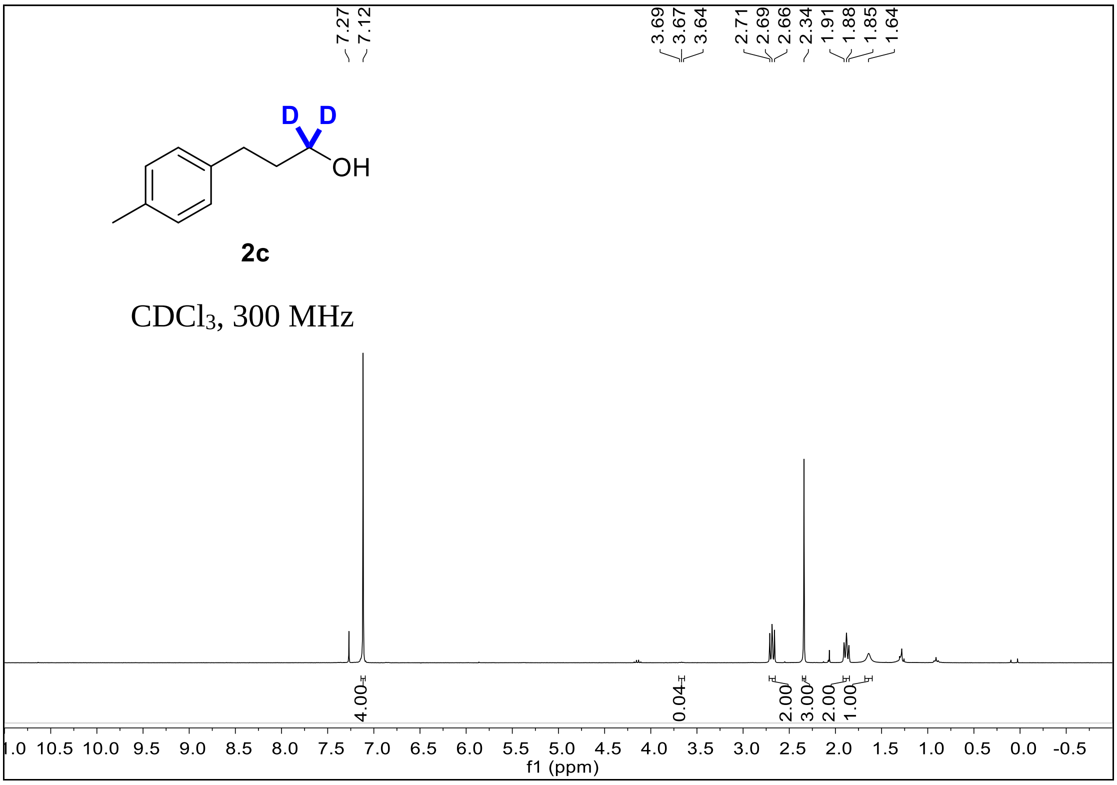
7.27
7.12
3.69
3.67
3.64
2.71
2.69
2.66
2.34
1.91
1.88
1.85
1.64
D
D
OH
2c
CDCl3, 300 MHz
4.00
0.04
2.00
3.00
2.00
1.00
f1 (ppm)

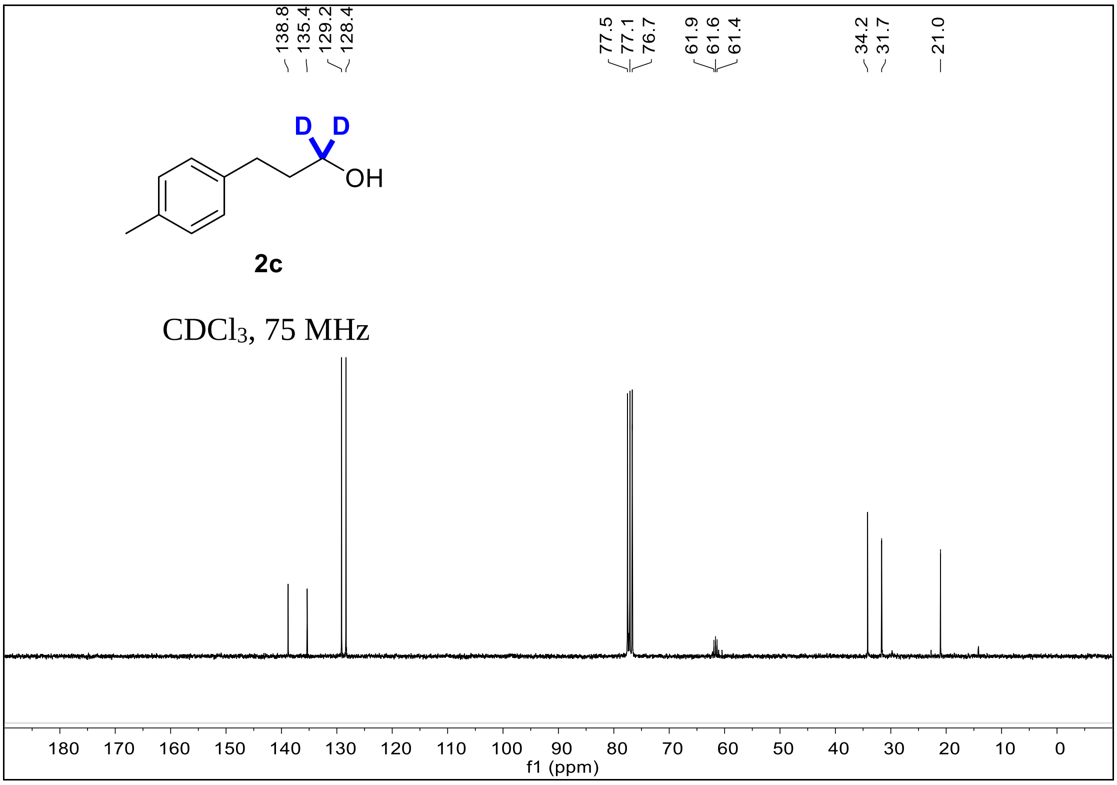
138.8
135.4
129.2
128.4
77.5
77.1
76.7
61.9
61.6
61.4
34.2
31.7
21.0
D
D
OH
2c
CDCl3, 75 MHz
f1 (ppm)

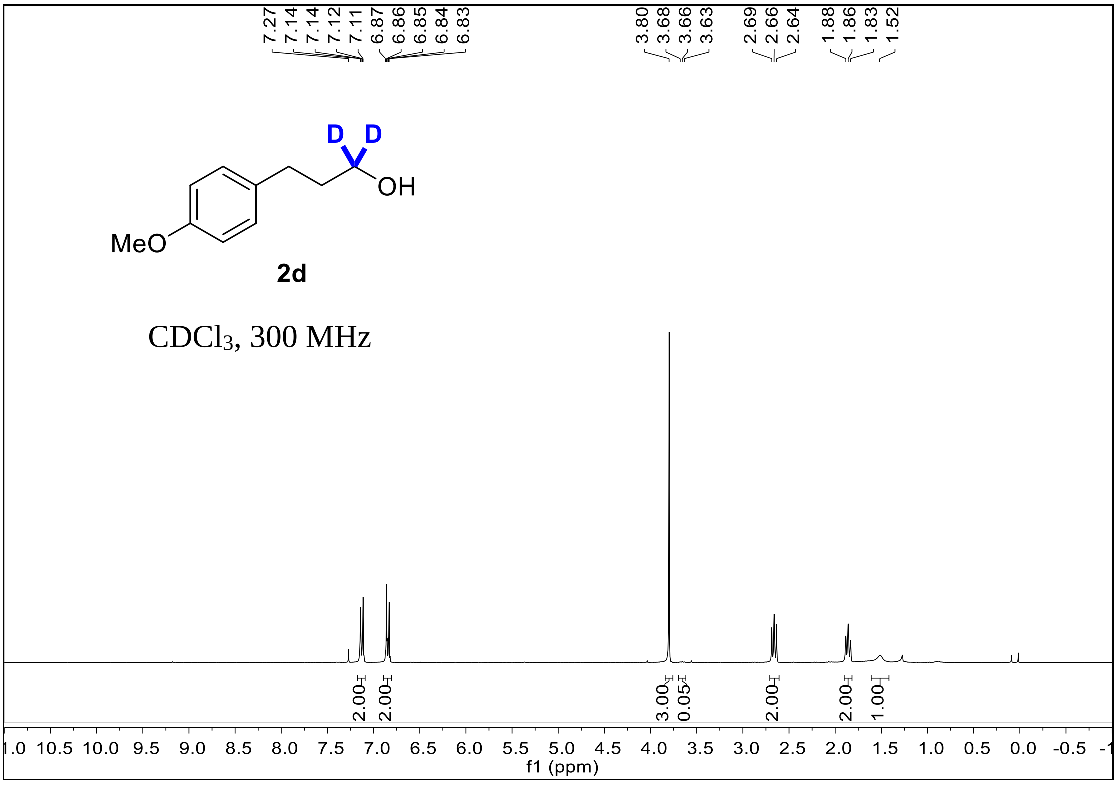
7.27
7.14
7.14
7.12
7.11
6.87
6.86
6.85
6.84
6.83
3.80
3.68
3.66
3.63
2.69
2.66
2.64
1.88
1.86
1.83
1.52
D
D
OH
MeO
2d
CDCl3, 300 MHz
2.00
2.00
3.00
0.05
2.00
2.00
1.00
f1 (ppm)

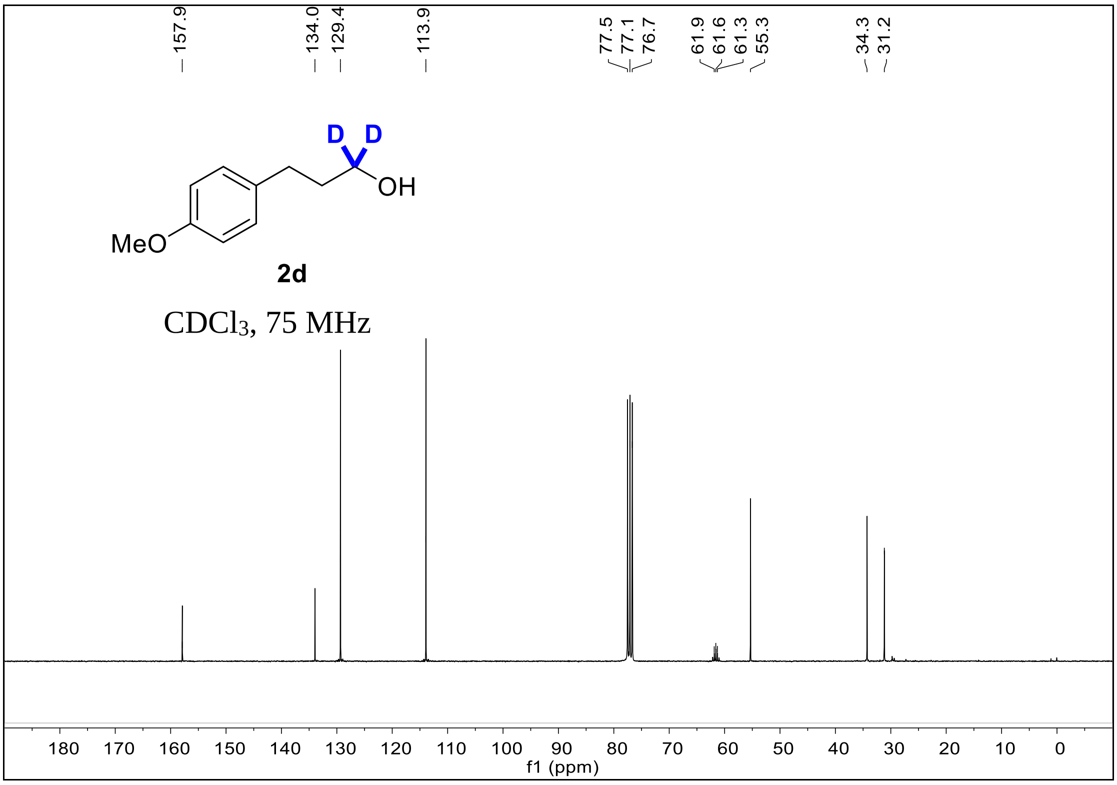
157.9
134.0
129.4
113.9
77.5
77.1
76.7
61.9
61.6
61.3
55.3
34.3
31.2
D
D
OH
MeO
2d
CDCl3, 75 MHz
f1 (ppm)

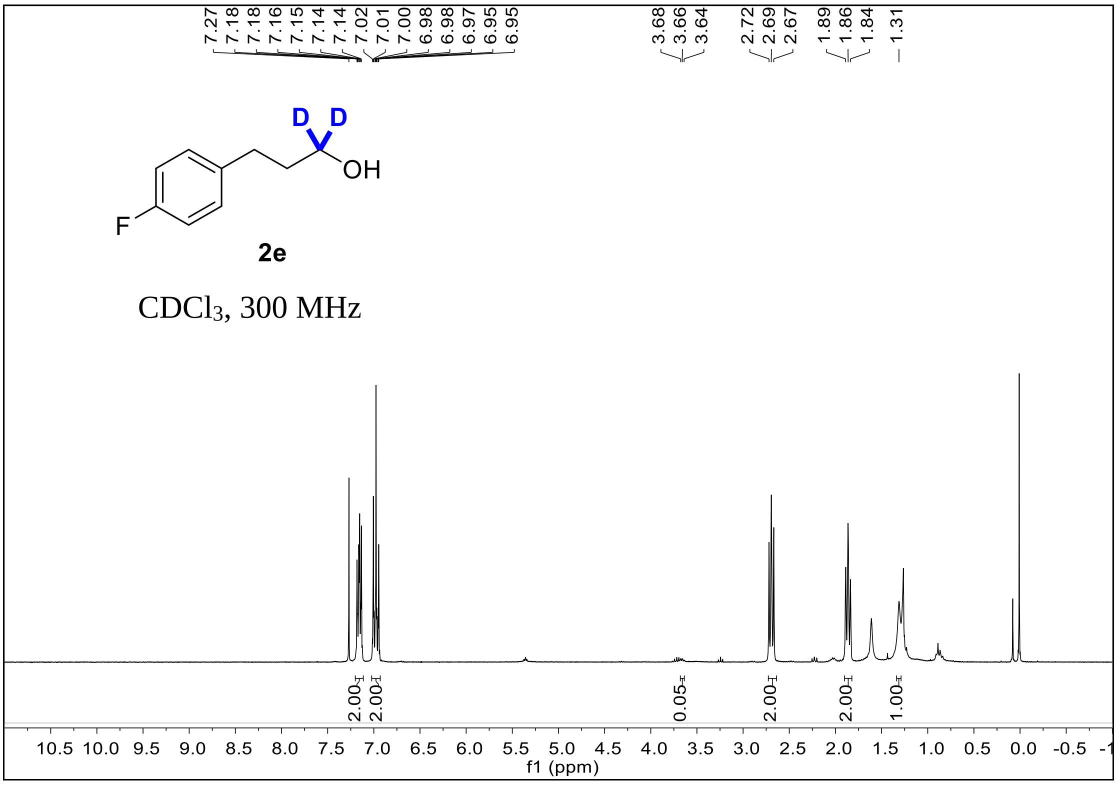
7.27
7.18
7.18
7.16
7.15
7.14
7.14
7.02
7.01
7.00
6.98
6.98
6.97
6.95
6.95
3.68
3.66
3.64
2.72
2.69
2.67
1.89
1.86
1.84
1.31
D D
OH
F
2e
CDCl3, 300 MHz
2.00
2.00
0.05
2.00
2.00
1.00
10.5 10.0 9.5 9.0 8.5 8.0 7.5 7.0 6.5 6.0 5.5 5.0 4.5 4.0 3.5 3.0 2.5 2.0 1.5 1.0 0.5 0.0 -0.5
f1 (ppm)

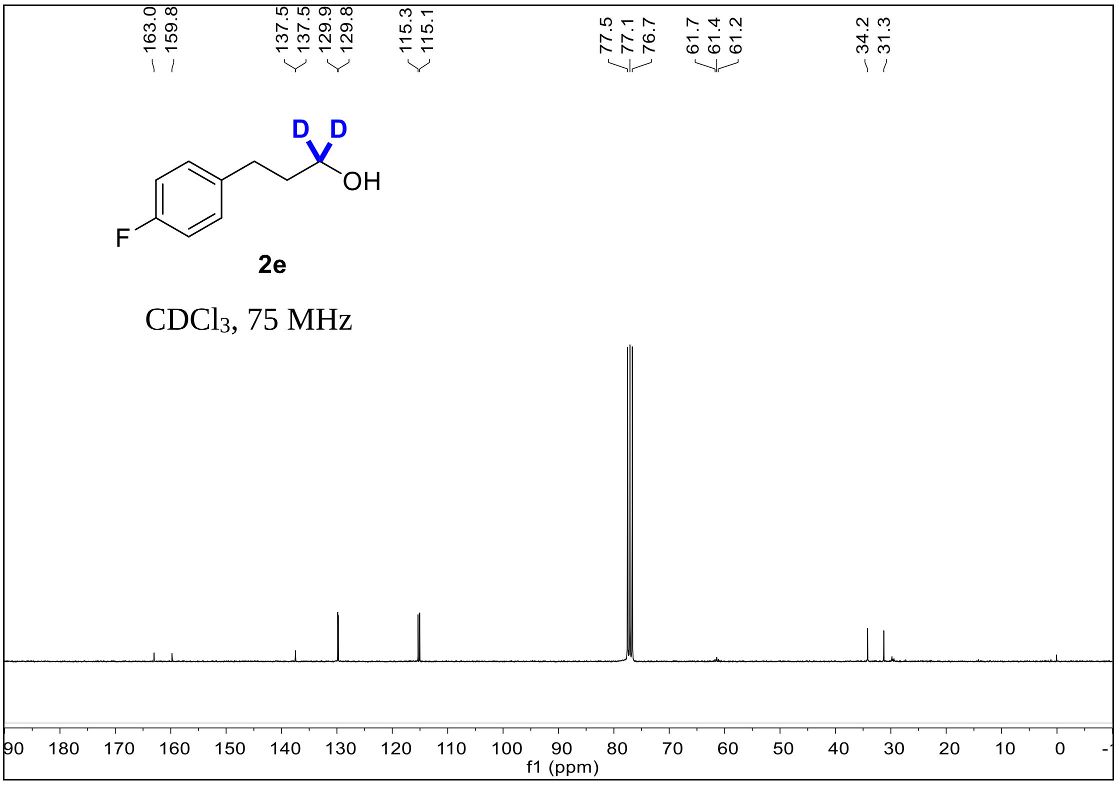
163.0
159.8
137.5
137.5
129.9
129.8
115.3
115.1
77.5
77.1
76.7
61.7
61.4
61.2
34.2
31.3
D D
OH
F
2e
CDCl3, 75 MHz
180 170 160 150 140 130 120 110 100 90 80 70 60 50 40 30 20 10 0
f1 (ppm)

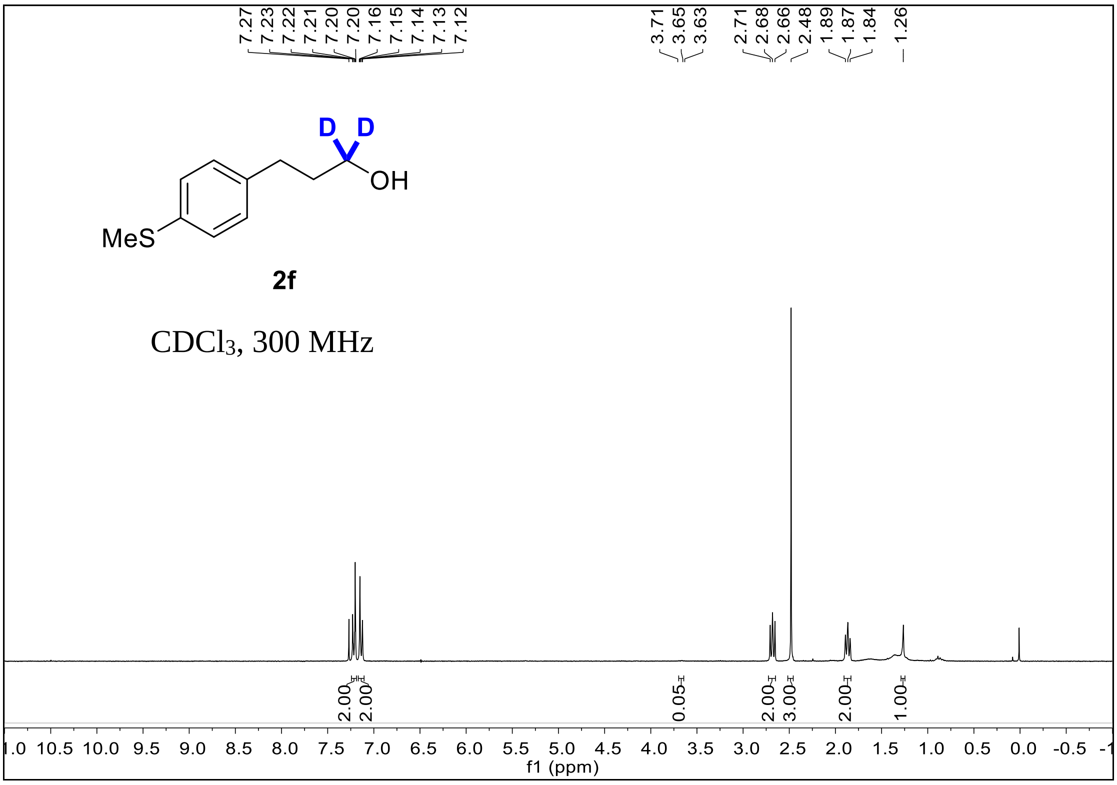
7.27 7.23 7.22 7.21 7.20 7.20 7.16 7.15 7.14 7.13 7.12
3.71 3.65 3.63
2.71 2.68 2.66 2.48 1.89 1.87 1.84
1.26
D D
OH
MeS
2f
CDCl3, 300 MHz
2.00 2.00 0.05 2.00 3.00 2.00 1.00
f1 (ppm)

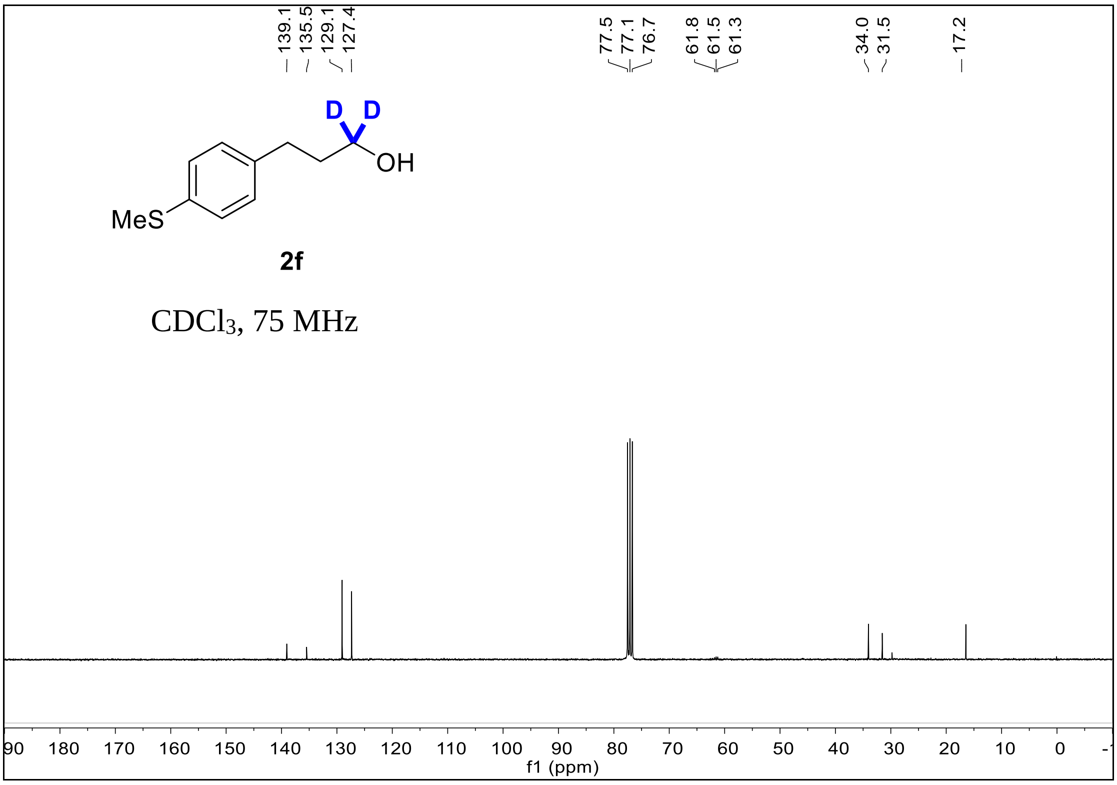
139.1 135.5 129.1 127.4
77.5 77.1 76.7
61.8 61.5 61.3
34.0 31.5
17.2
D D
OH
MeS
2f
CDCl3, 75 MHz
f1 (ppm)

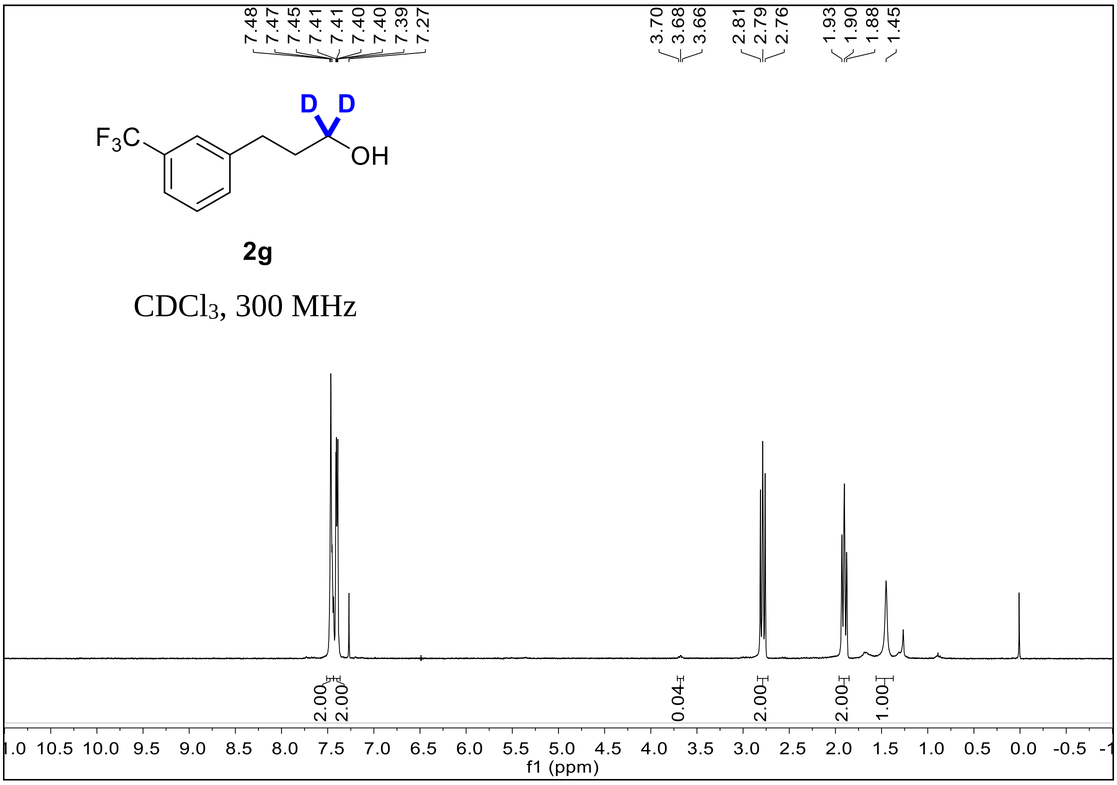
7.48
7.47
7.45
7.41
7.41
7.40
7.40
7.39
7.27
3.70
3.68
3.66
2.81
2.79
2.76
1.93
1.90
1.88
1.45
D
D
F3C
OH
2g
CDCl3, 300 MHz
2.00
2.00
0.04
2.00
2.00
1.00
f1 (ppm)

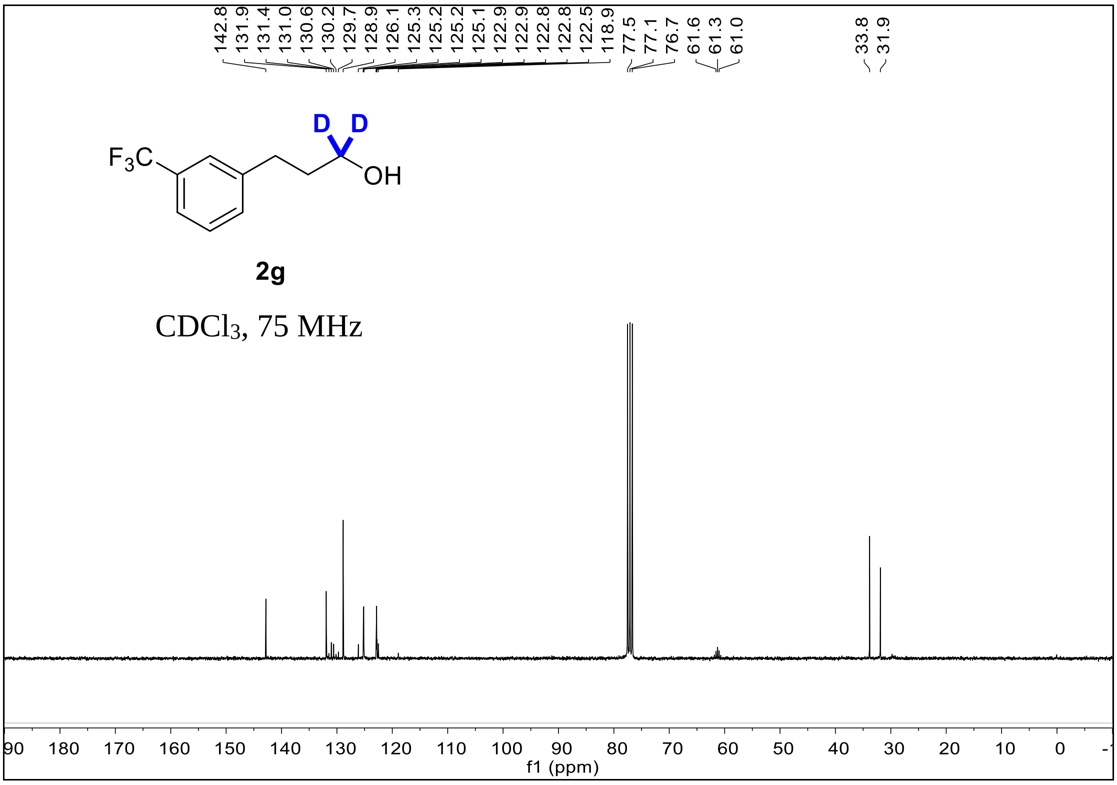
142.8
131.9
131.4
131.0
130.6
130.2
129.7
128.9
126.1
125.3
125.2
125.2
125.1
122.9
122.9
122.8
122.8
122.5
118.9
77.5
77.1
76.7
61.6
61.3
61.0
33.8
31.9
D
D
F3C
OH
2g
CDCl3, 75 MHz
f1 (ppm)

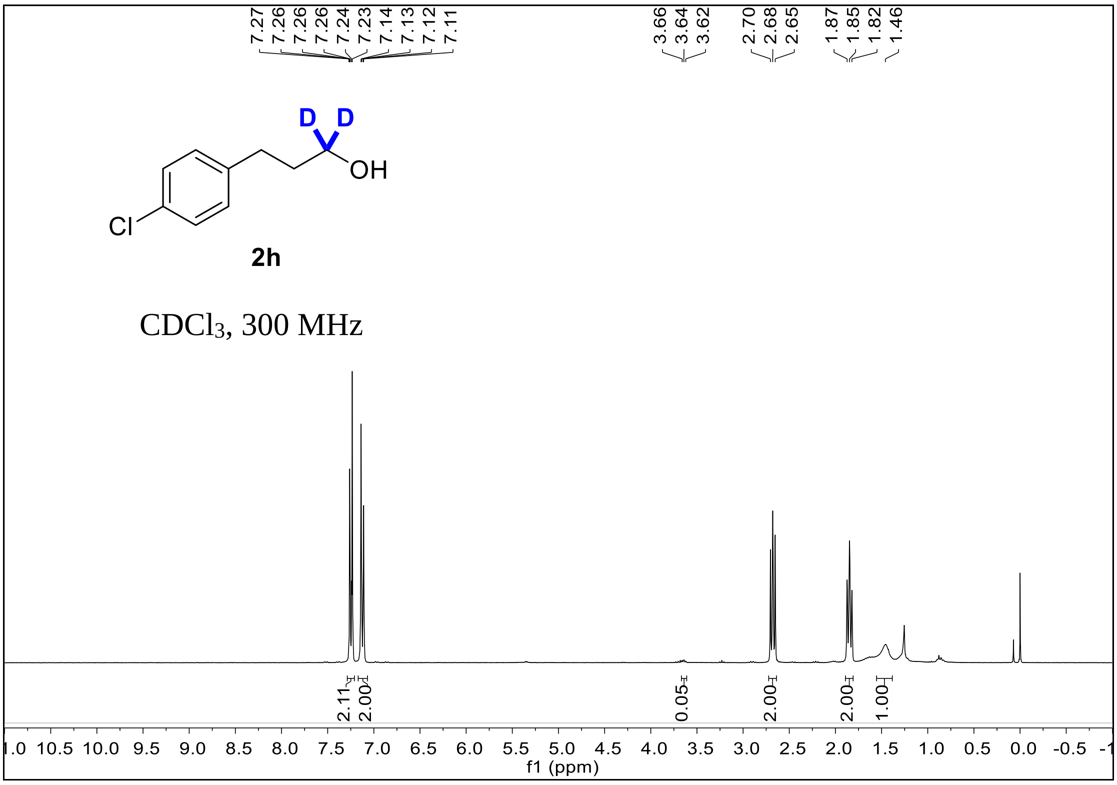
7.27
7.26
7.26
7.26
7.24
7.23
7.14
7.13
7.12
7.11
3.66
3.64
3.62
2.70
2.68
2.65
1.87
1.85
1.82
1.46
D
D
OH
Cl
2h
CDCl3, 300 MHz
2.11
2.00
0.05
2.00
2.00
1.00
10.5 10.0 9.5 9.0 8.5 8.0 7.5 7.0 6.5 6.0 5.5 5.0 4.5 4.0 3.5 3.0 2.5 2.0 1.5 1.0 0.5 0.0 -0.5
f1 (ppm)

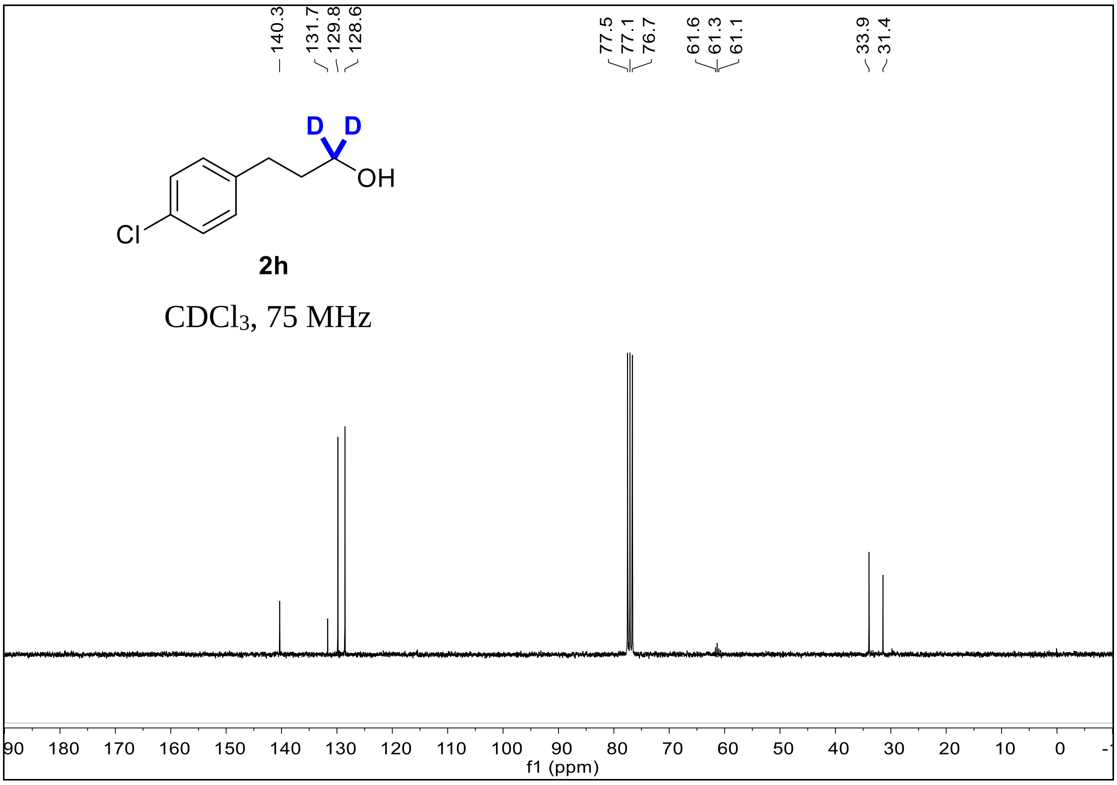
140.3
131.7
129.8
128.6
77.5
77.1
76.7
61.6
61.3
61.1
33.9
31.4
D
D
OH
Cl
2h
CDCl3, 75 MHz
180 170 160 150 140 130 120 110 100 90 80 70 60 50 40 30 20 10 0
f1 (ppm)

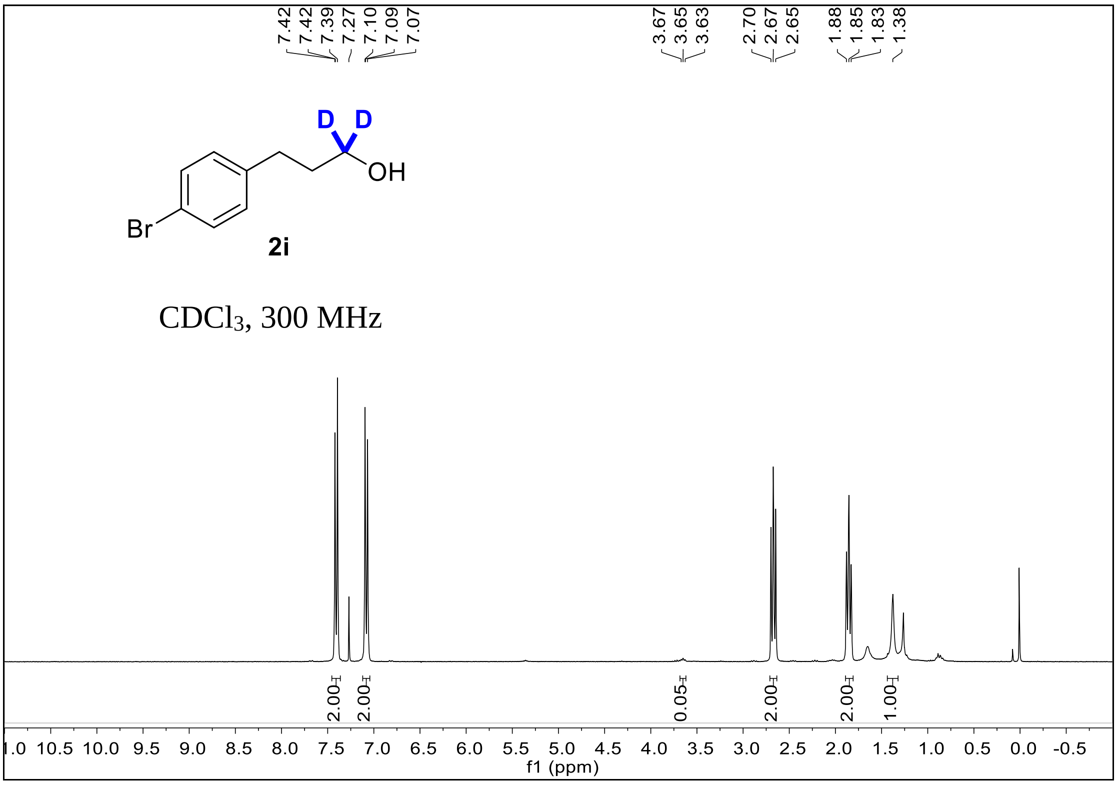
7.42 7.42 7.39 7.27 7.10 7.09 7.07
3.67 3.65 3.63
2.70 2.67 2.65
1.88 1.85 1.83 1.38
D D
OH
Br
2i
CDCl3, 300 MHz
2.00 2.00 0.05 2.00 2.00 1.00
f1 (ppm)

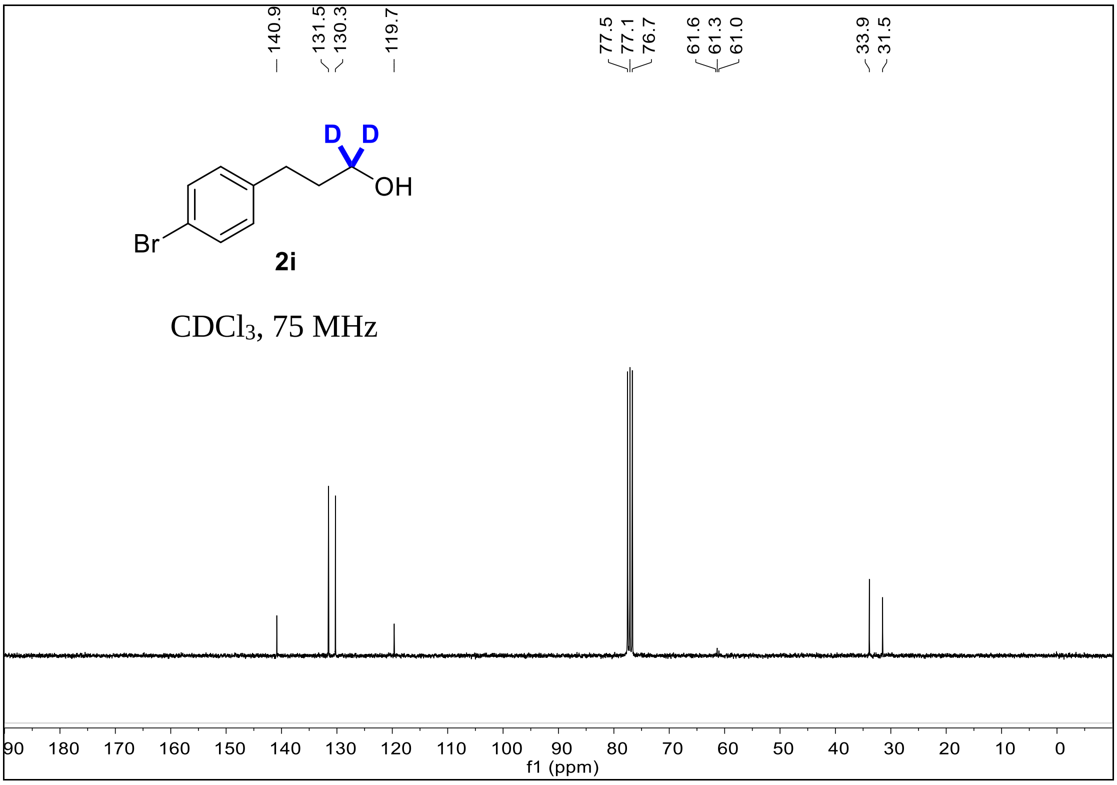
140.9 131.5 130.3 119.7
77.5 77.1 76.7
61.6 61.3 61.0
33.9 31.5
D D
OH
Br
2i
CDCl3, 75 MHz
f1 (ppm)

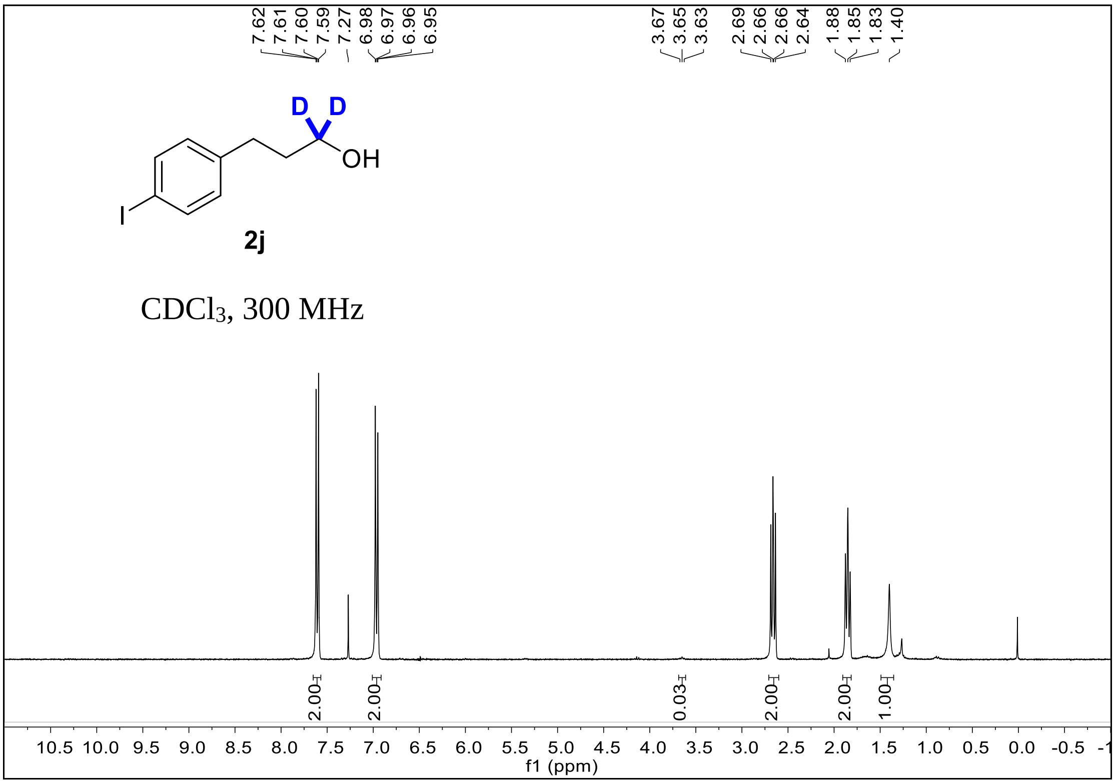

7.62 7.61 7.60 7.59 7.27 6.98 6.97 6.96 6.95
3.67 3.65 3.63 2.69 2.66 2.66 2.64 1.88 1.85 1.83 1.40
D D
OH
I
2j
CDCl3, 300 MHz
2.00 2.00 0.03 2.00 2.00 1.00
10.5 10.0 9.5 9.0 8.5 8.0 7.5 7.0 6.5 6.0 5.5 5.0 4.5 4.0 3.5 3.0 2.5 2.0 1.5 1.0 0.5 0.0 -0.5
f1 (ppm)

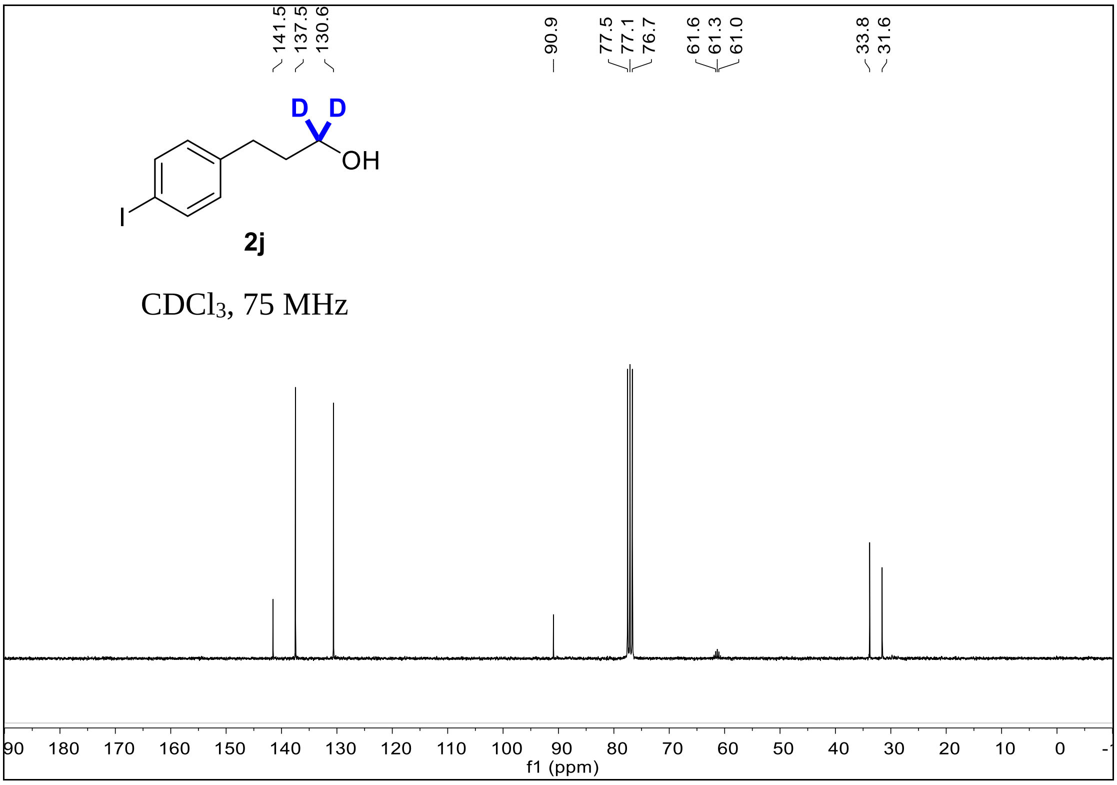

141.5 137.5 130.6
90.9
77.5 77.1 76.7
61.6 61.3 61.0
33.8 31.6
D D
OH
I
2j
CDCl3, 75 MHz
180 170 160 150 140 130 120 110 100 90 80 70 60 50 40 30 20 10 0
f1 (ppm)

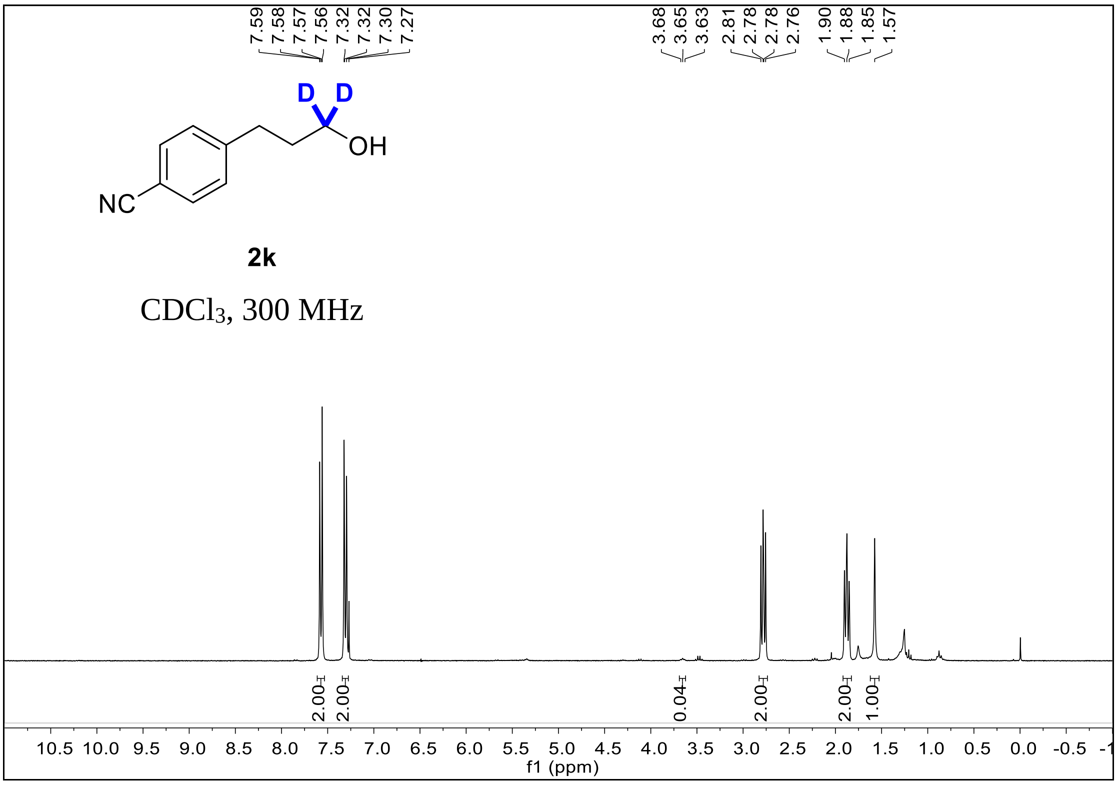
7.59 7.58 7.57 7.56 7.32 7.32 7.30 7.27
3.68 3.65 3.63 2.81 2.78 2.78 2.76 1.90 1.88 1.85 1.57
D D
OH
NC
2k
CDCl3, 300 MHz
2.00 2.00 0.04 2.00 2.00 1.00
10.5 10.0 9.5 9.0 8.5 8.0 7.5 7.0 6.5 6.0 5.5 5.0 4.5 4.0 3.5 3.0 2.5 2.0 1.5 1.0 0.5 0.0 -0.5
f1 (ppm)

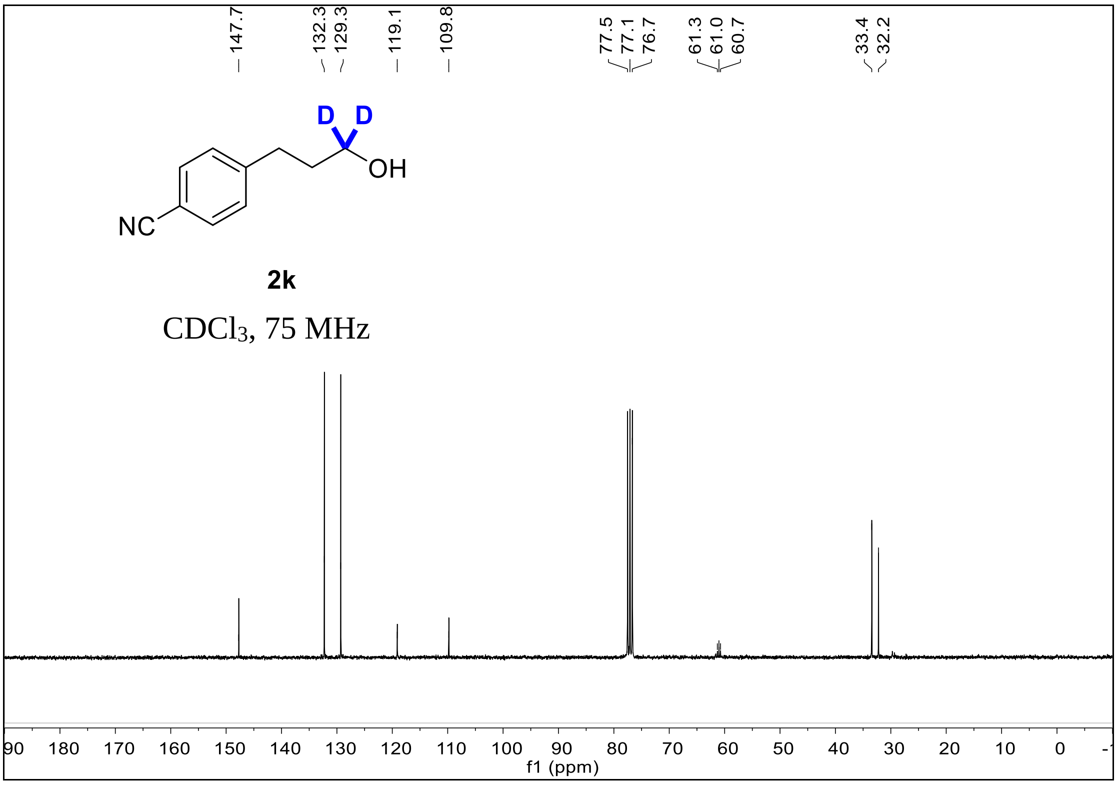
147.7 132.3 129.3 119.1 109.8 77.5 77.1 76.7 61.3 61.0 60.7 33.4 32.2
D D
OH
NC
2k
CDCl3, 75 MHz
180 170 160 150 140 130 120 110 100 90 80 70 60 50 40 30 20 10 0
f1 (ppm)

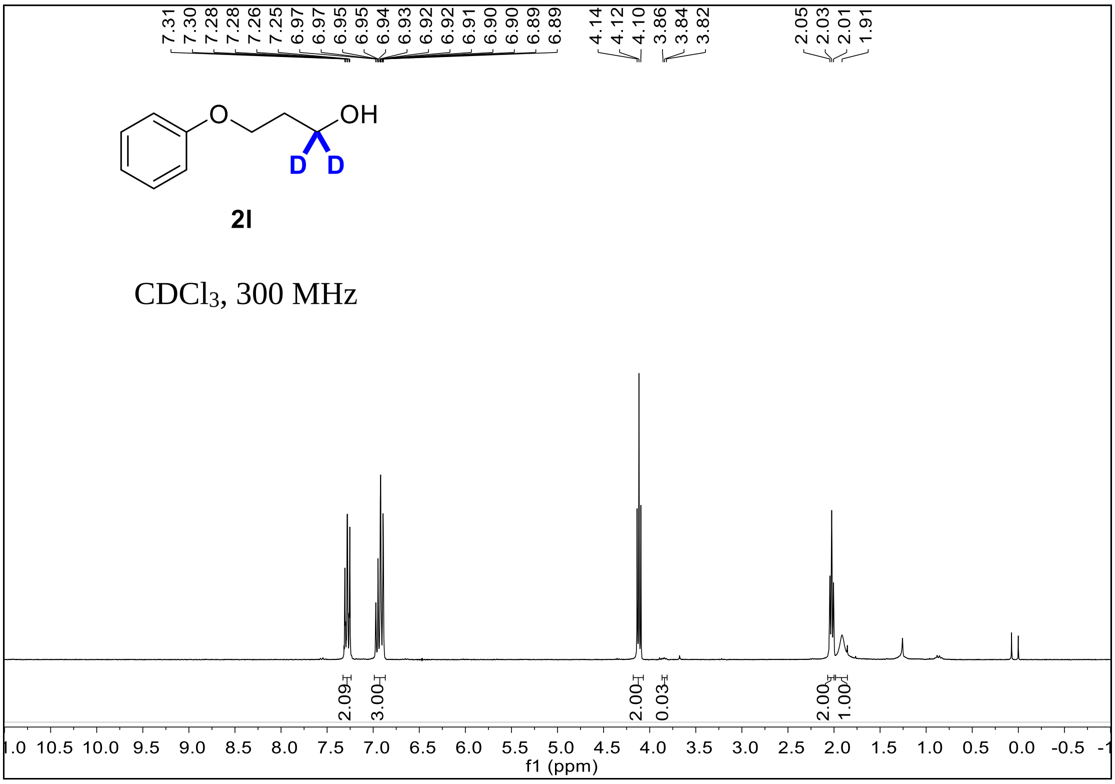
7.31 7.30 7.28 7.28 7.26 7.25 6.97 6.97 6.95 6.95 6.94 6.93 6.92 6.92 6.91 6.90 6.90 6.89 6.89
4.14 4.12 4.10 3.86 3.84 3.82
2.05 2.03 2.01 1.91
O
OH
D D
2l
CDCl3, 300 MHz
2.09 3.00 2.00 0.03 2.00 1.00
f1 (ppm)

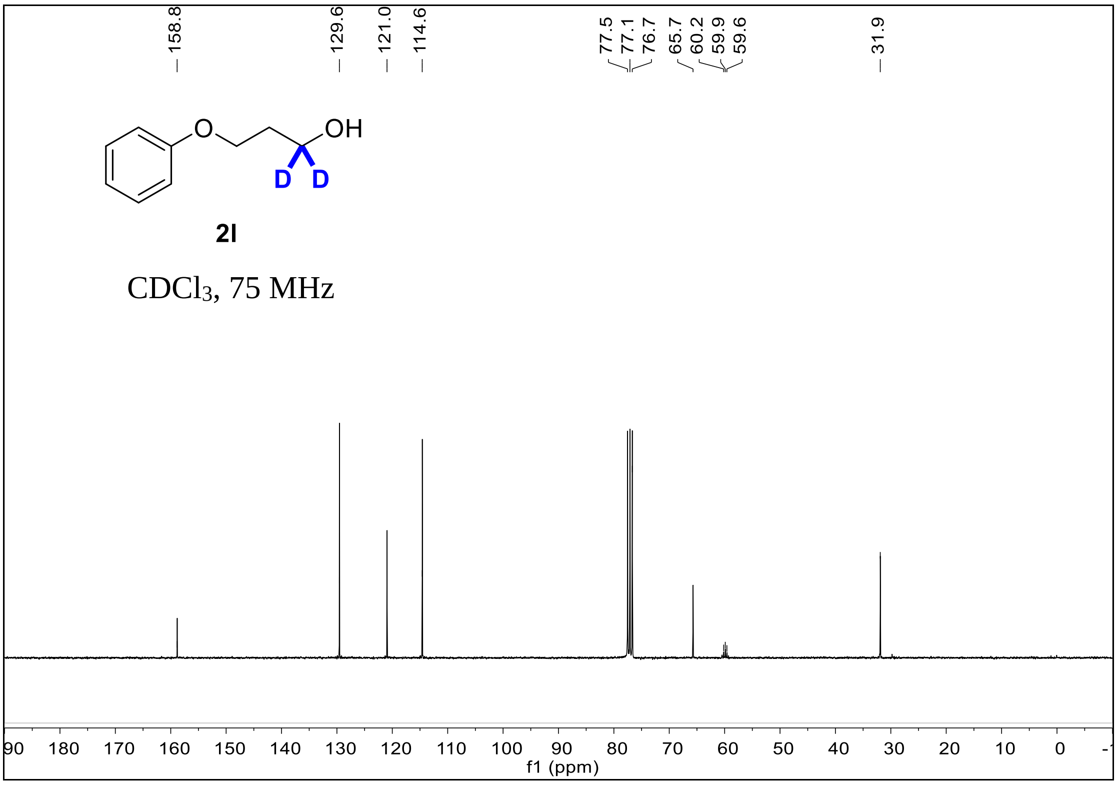
158.8
129.6 121.0 114.6
77.5 77.1 76.7 65.7 60.2 59.9 59.6
31.9
O
OH
D D
2l
CDCl3, 75 MHz
f1 (ppm)

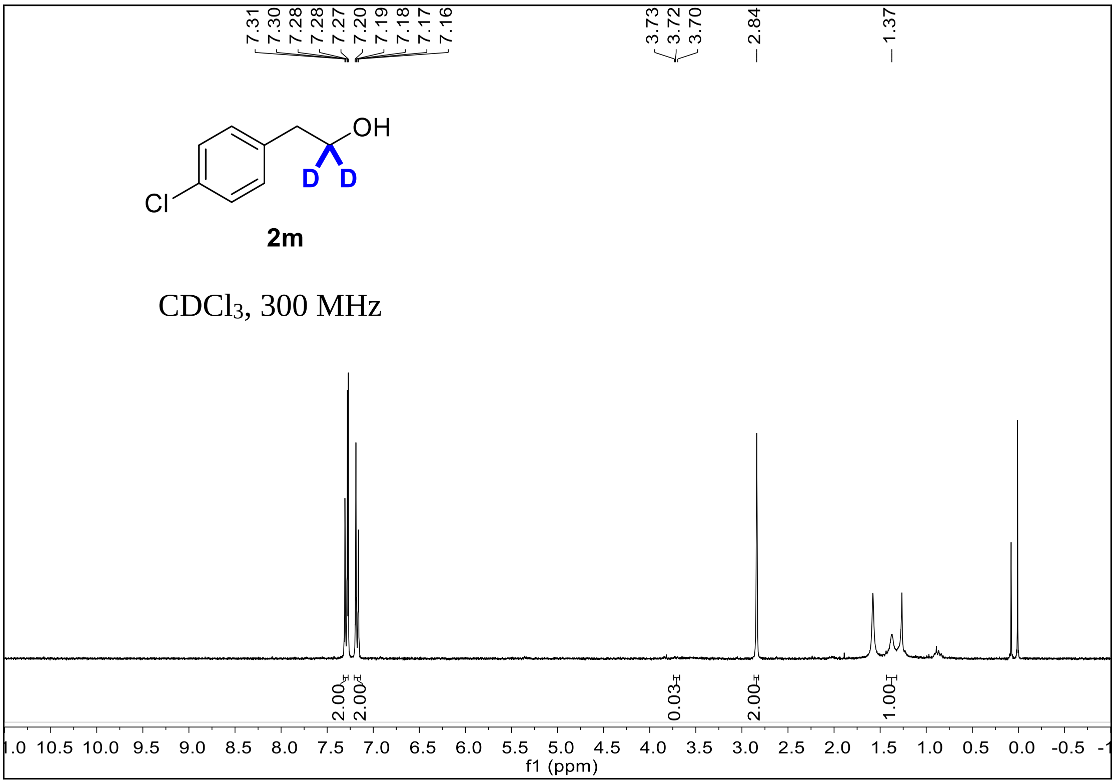
7.31
7.30
7.28
7.28
7.27
7.20
7.19
7.18
7.17
7.16
3.73
3.72
3.70
2.84
1.37
OH
D D
Cl
2m
CDCl3, 300 MHz
2.00
2.00
0.03
2.00
1.00
f1 (ppm)

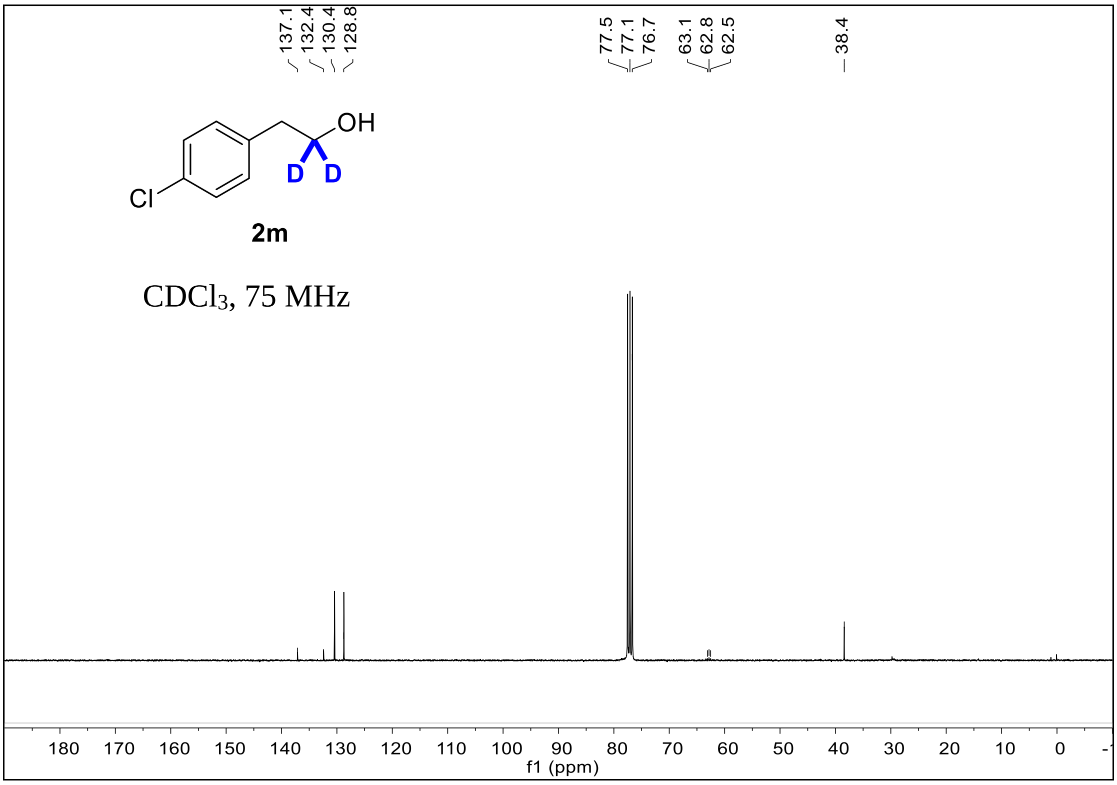
137.1
132.4
130.4
128.8
77.5
77.1
76.7
63.1
62.8
62.5
38.4
OH
D D
Cl
2m
CDCl3, 75 MHz
f1 (ppm)

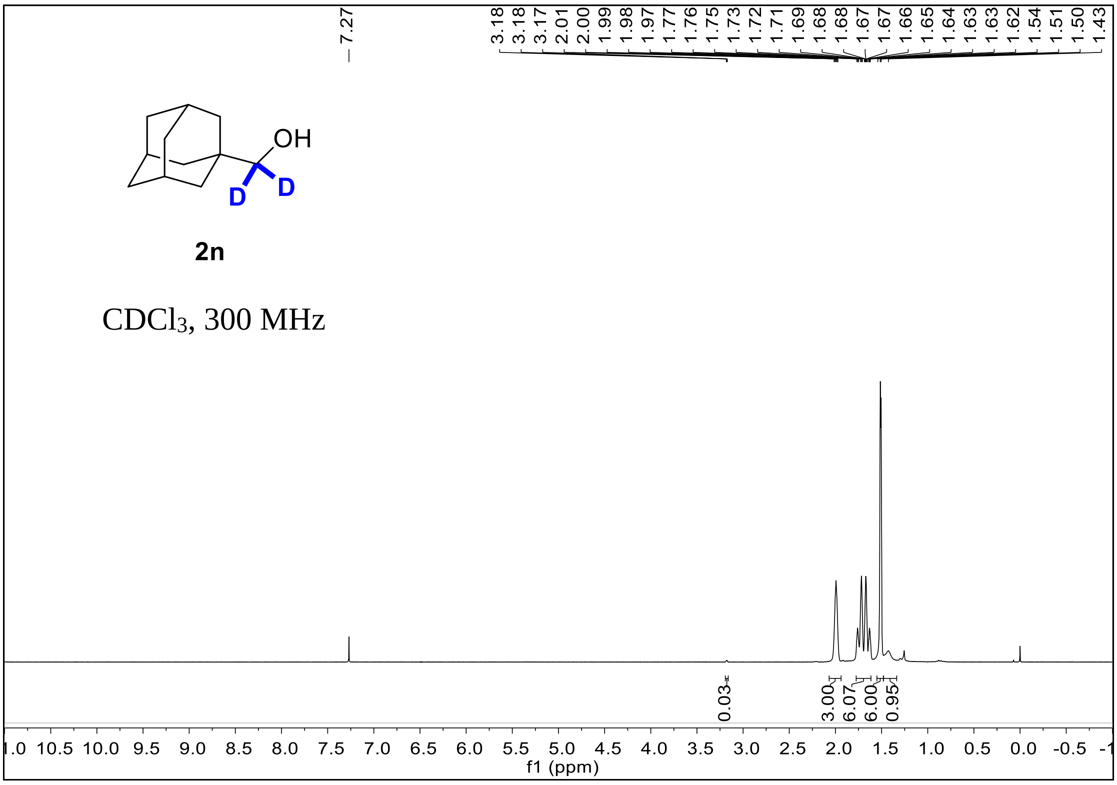

7.27
3.18
3.18
3.17
2.01
2.00
1.99
1.98
1.97
1.77
1.76
1.75
1.73
1.72
1.71
1.69
1.68
1.68
1.67
1.67
1.66
1.65
1.64
1.63
1.63
1.62
1.54
1.51
1.50
1.43
OH
D
D
2n
CDCl3, 300 MHz
0.03
3.00
6.07
6.00
0.95
f1 (ppm)

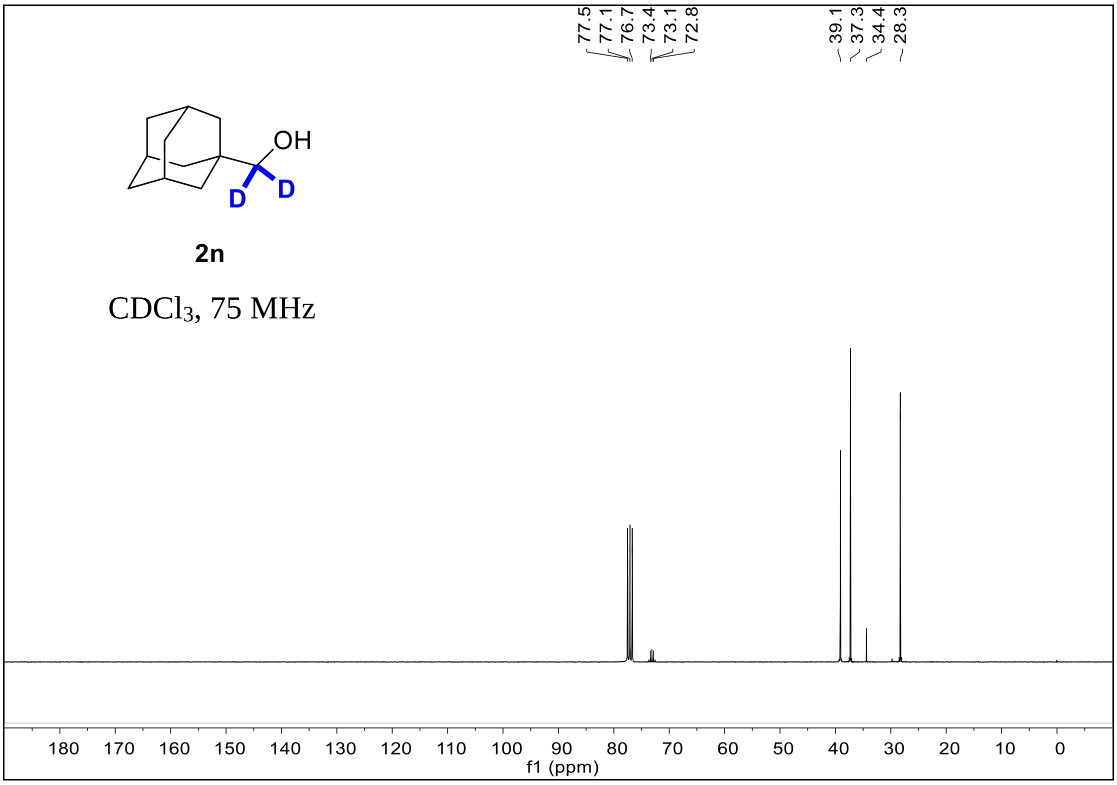

77.5
77.1
76.7
73.4
73.1
72.8
39.1
37.3
34.4
28.3
OH
D
D
2n
CDCl3, 75 MHz
f1 (ppm)

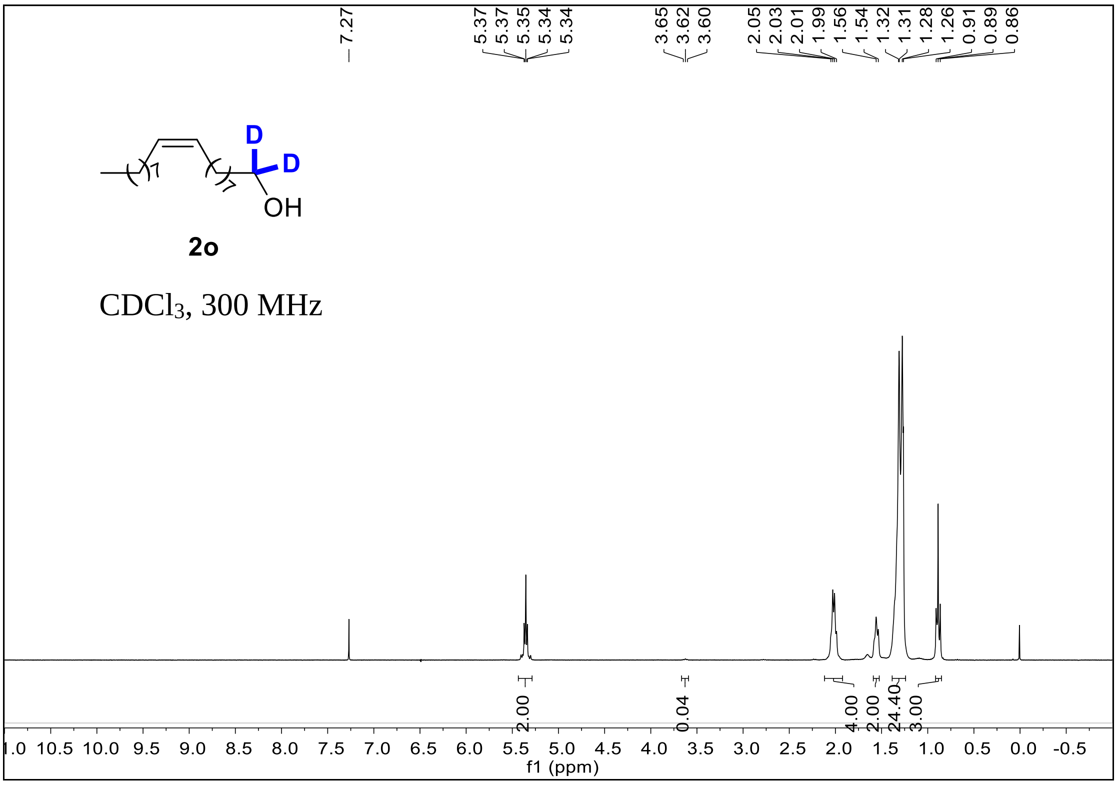
7.27
5.37
5.37
5.35
5.34
5.34
3.65
3.62
3.60
2.05
2.03
2.01
1.99
1.56
1.54
1.32
1.31
1.28
1.26
0.91
0.89
0.86
D
D
OH
2o
CDCl3, 300 MHz
2.00
0.04
4.00
2.00
24.40
3.00
1.0 10.5 10.0 9.5 9.0 8.5 8.0 7.5 7.0 6.5 6.0 5.5 5.0 4.5 4.0 3.5 3.0 2.5 2.0 1.5 1.0 0.5 0.0 -0.5
f1 (ppm)

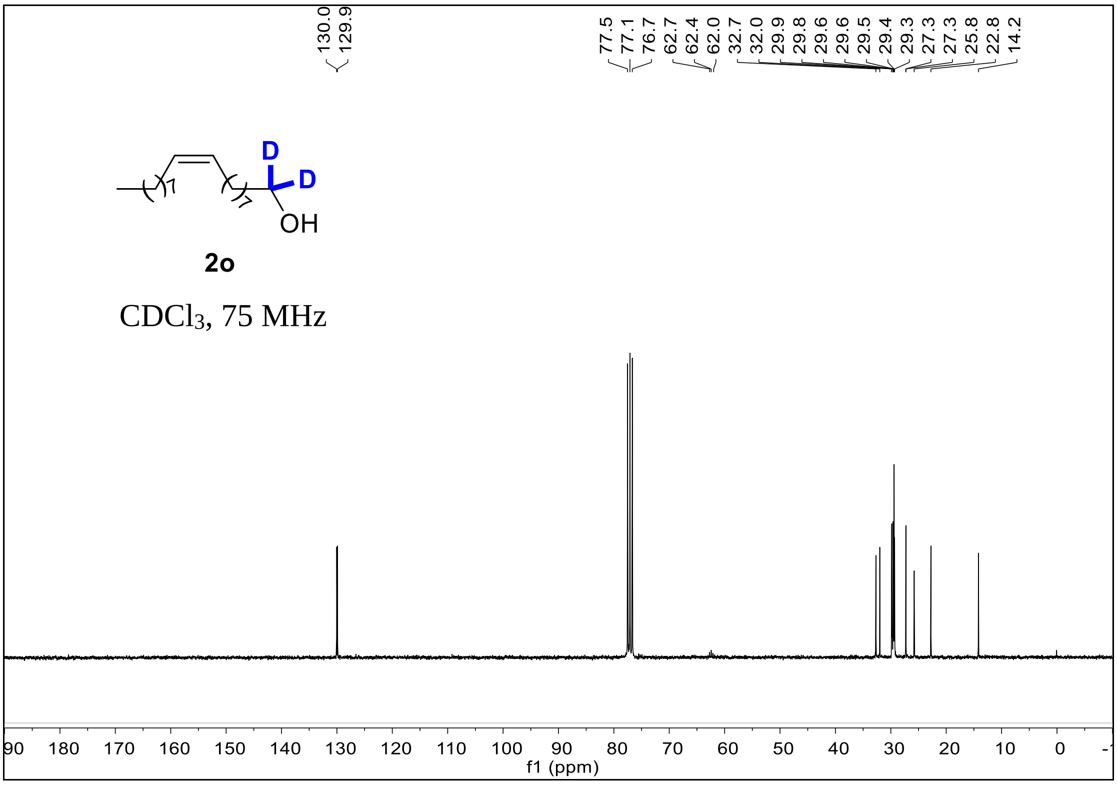
130.0
129.9
77.5
77.1
76.7
62.7
62.4
62.0
32.7
32.0
29.9
29.8
29.6
29.6
29.5
29.4
29.3
27.3
27.3
25.8
22.8
14.2
D
D
OH
2o
CDCl3, 75 MHz
90 180 170 160 150 140 130 120 110 100 90 80 70 60 50 40 30 20 10 0
f1 (ppm)

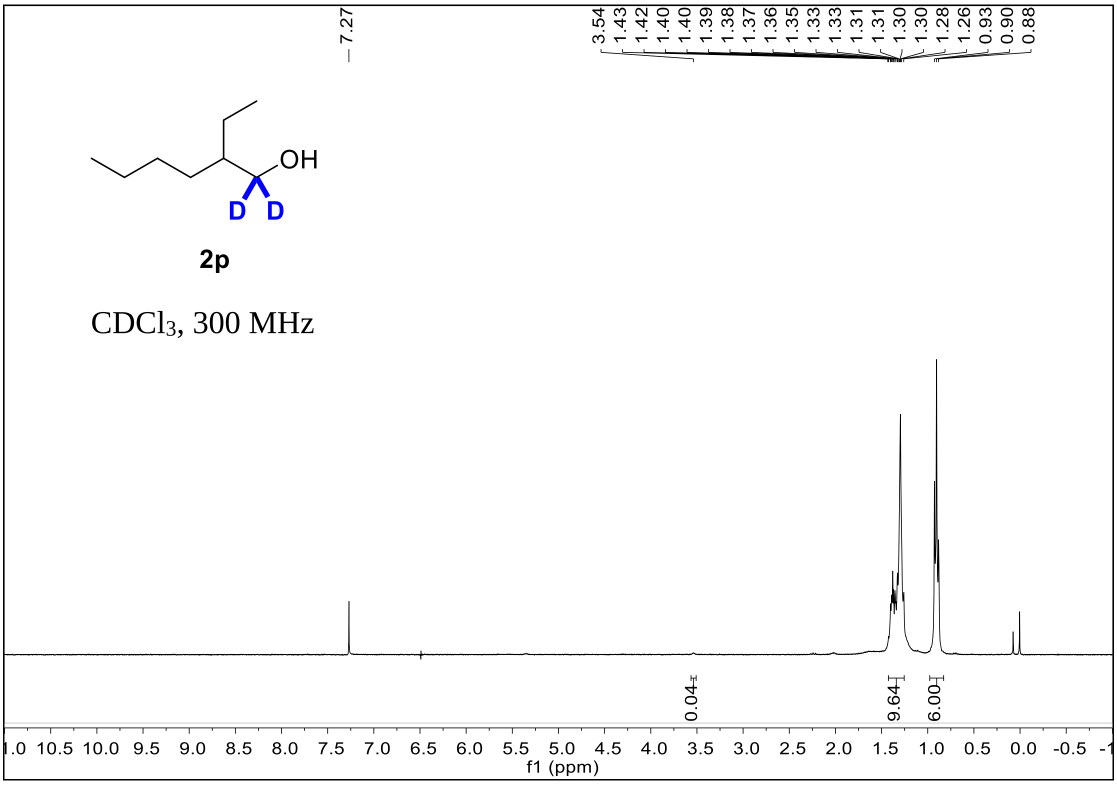

OH
D D
2p
CDCl3, 300 MHz
7.27
3.54 1.43 1.42 1.40 1.40 1.39 1.38 1.37 1.36 1.35 1.33 1.33 1.31 1.31 1.30 1.30 1.28 1.26 0.93 0.90 0.88
0.04
9.64
6.00
f1 (ppm)

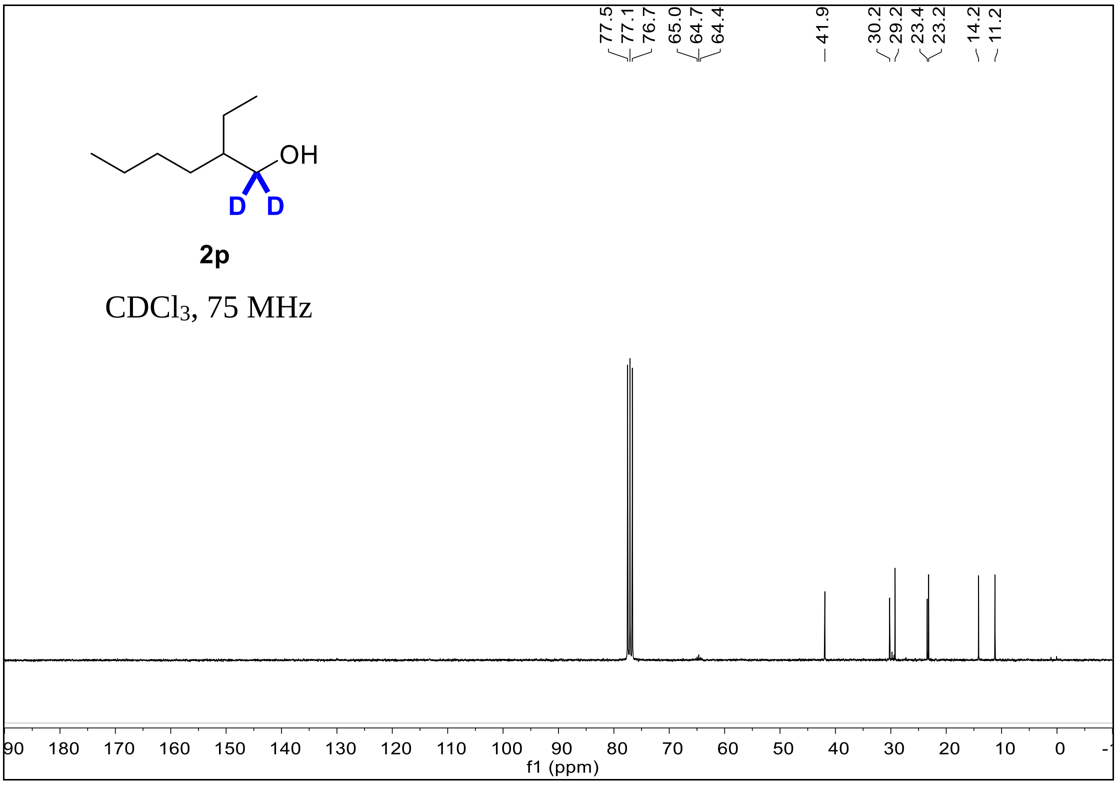

OH
D D
2p
CDCl3, 75 MHz
77.5 77.1 76.7 65.0 64.7 64.4
41.9
30.2 29.2 23.4 23.2 14.2 11.2
f1 (ppm)

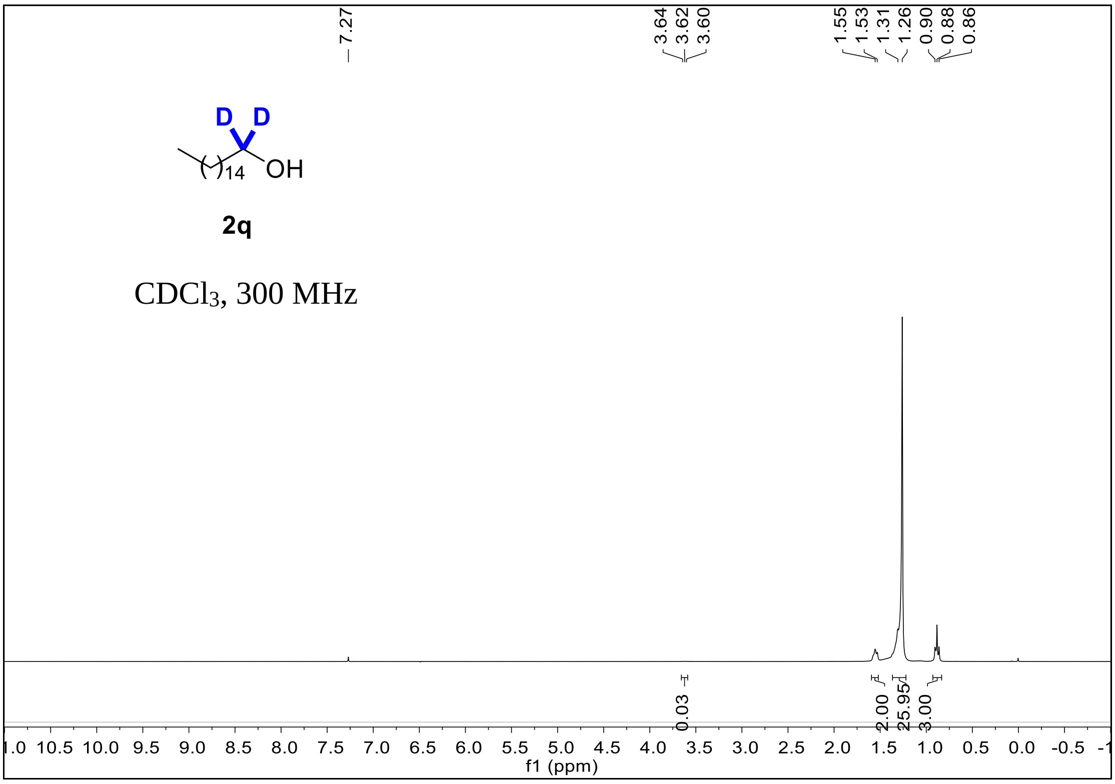
7.27
3.64
3.62
3.60
1.55
1.53
1.31
1.26
0.90
0.88
0.86
D D
OH
14
2q
CDCl3, 300 MHz
0.03
2.00
25.95
3.00
f1 (ppm)

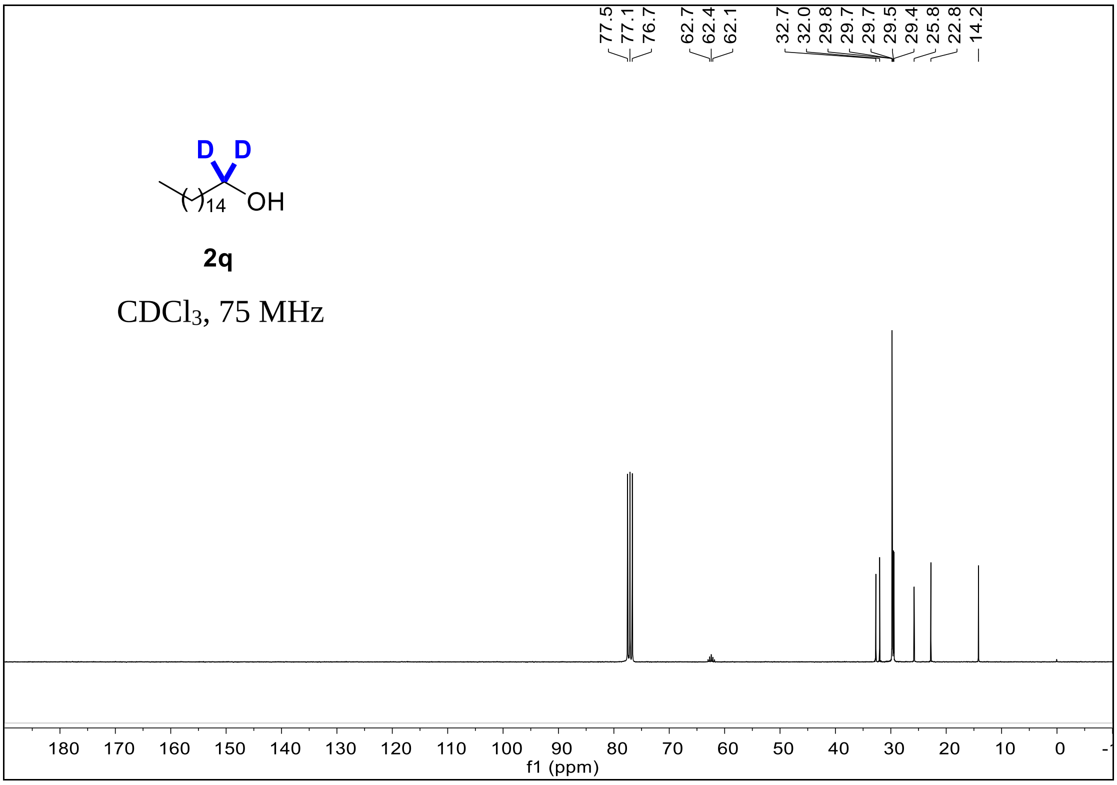
77.5
77.1
76.7
62.7
62.4
62.1
32.7
32.0
29.8
29.7
29.7
29.5
29.4
25.8
22.8
14.2
D D
OH
14
2q
CDCl3, 75 MHz
f1 (ppm)

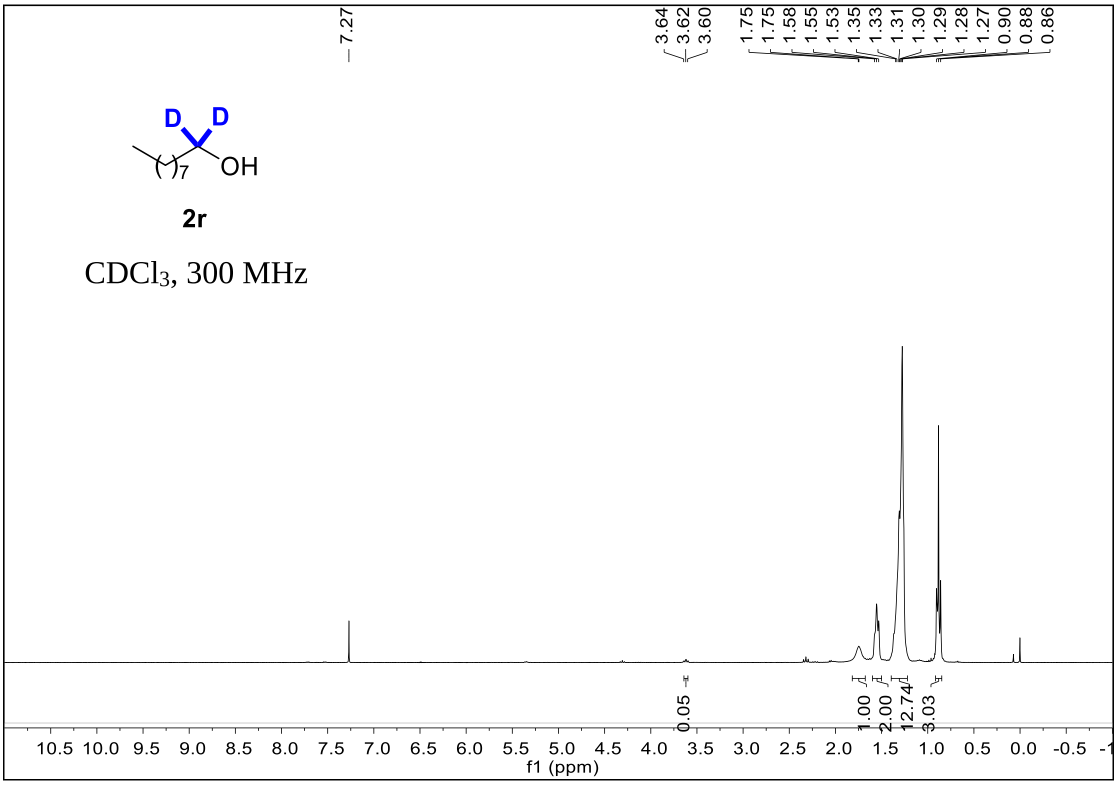
7.27
3.64
3.62
3.60
1.75
1.75
1.58
1.55
1.53
1.35
1.33
1.31
1.30
1.29
1.28
1.27
0.90
0.88
0.86
D
D
7
OH
2r
CDCl3, 300 MHz
0.05
1.00
2.00
12.74
3.03
10.5 10.0 9.5 9.0 8.5 8.0 7.5 7.0 6.5 6.0 5.5 5.0 4.5 4.0 3.5 3.0 2.5 2.0 1.5 1.0 0.5 0.0 -0.5
f1 (ppm)

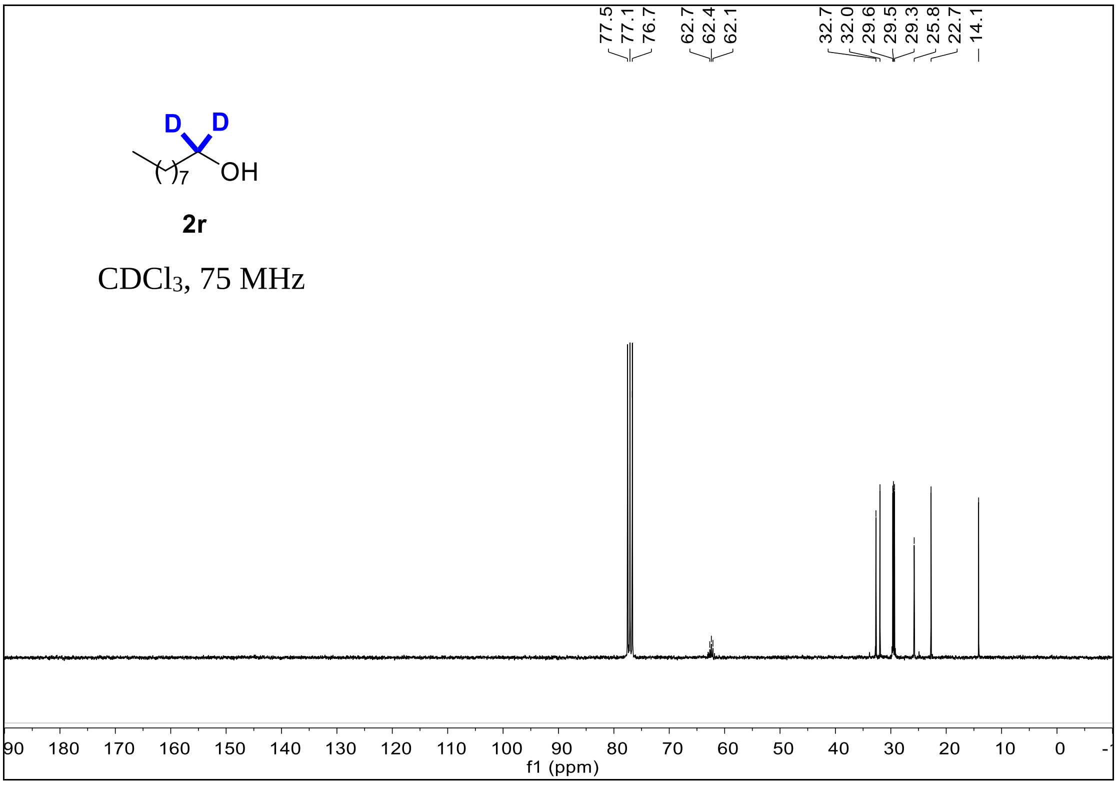
77.5
77.1
76.7
62.7
62.4
62.1
32.7
32.0
29.6
29.5
29.3
25.8
22.7
14.1
D
D
7
OH
2r
CDCl3, 75 MHz
180 170 160 150 140 130 120 110 100 90 80 70 60 50 40 30 20 10 0
f1 (ppm)

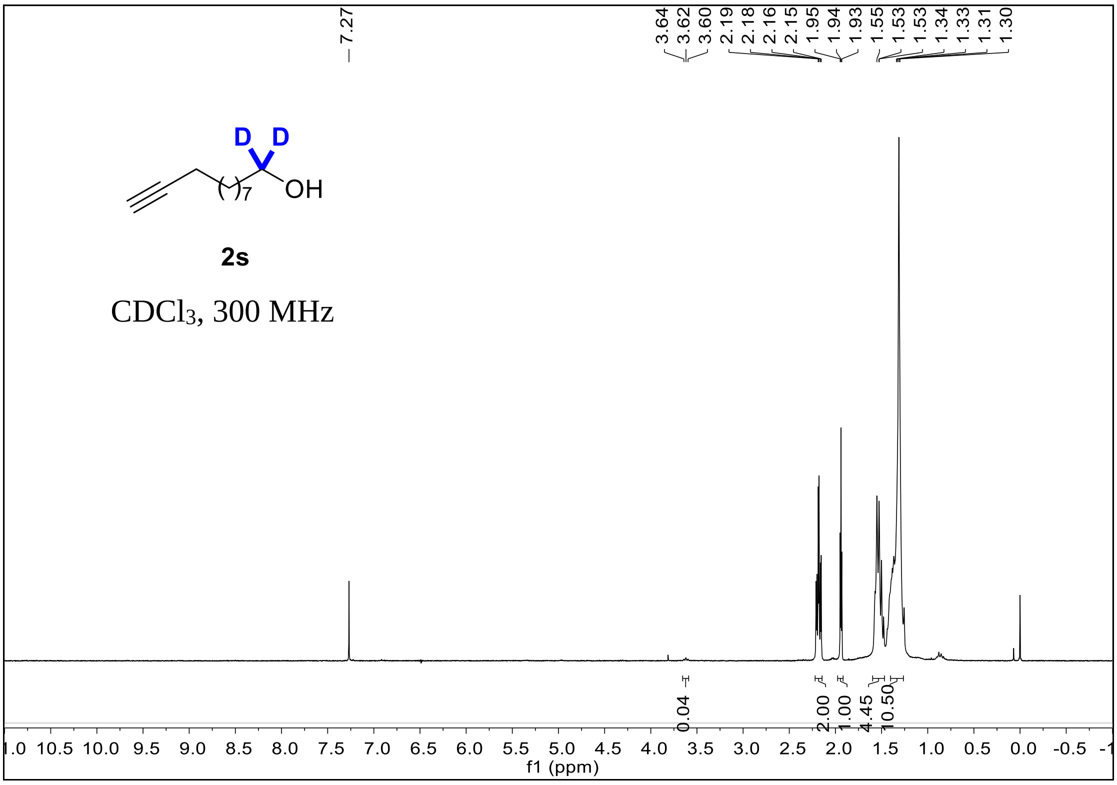

7.27
3.64
3.62
3.60
2.19
2.18
2.16
2.15
1.95
1.94
1.93
1.55
1.53
1.53
1.34
1.33
1.31
1.30
D D
OH
7
2s
CDCl3, 300 MHz
0.04
2.00
1.00
4.45
10.50
11.0 10.5 10.0 9.5 9.0 8.5 8.0 7.5 7.0 6.5 6.0 5.5 5.0 4.5 4.0 3.5 3.0 2.5 2.0 1.5 1.0 0.5 0.0 -0.5
f1 (ppm)

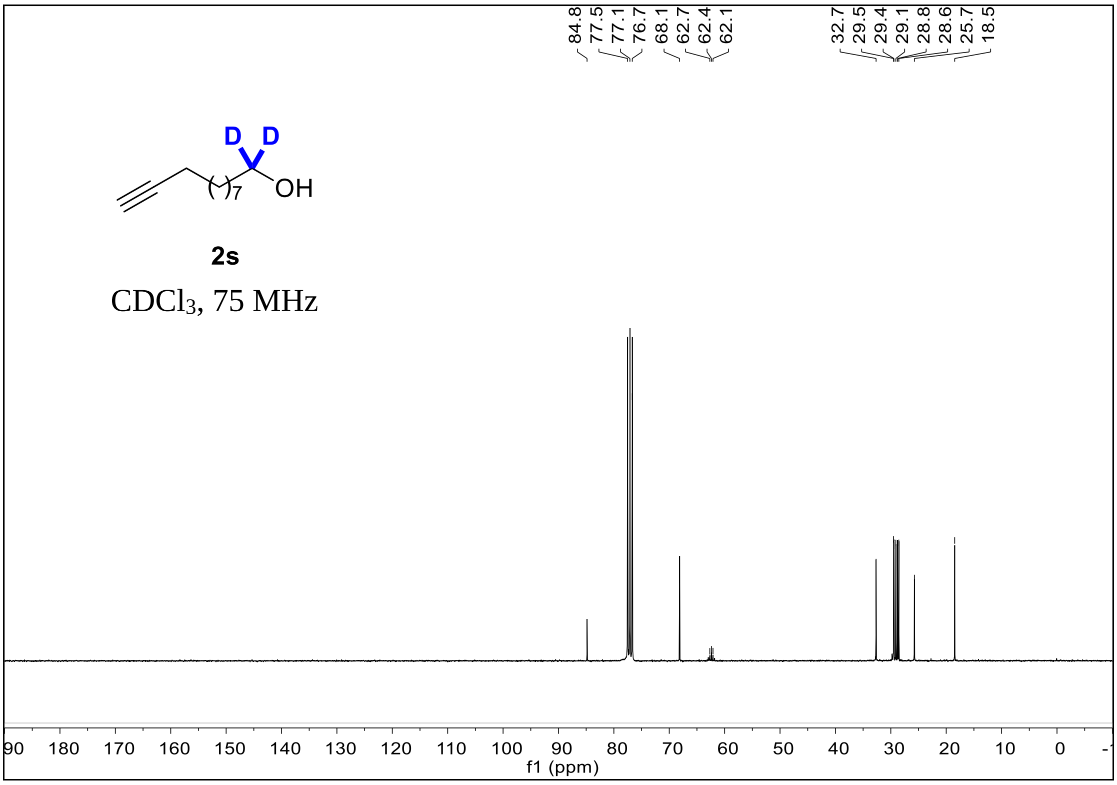

84.8
77.5
77.1
76.7
68.1
62.7
62.4
62.1
32.7
29.5
29.4
29.1
28.8
28.6
25.7
18.5
D D
OH
7
2s
CDCl3, 75 MHz
180 170 160 150 140 130 120 110 100 90 80 70 60 50 40 30 20 10 0
f1 (ppm)

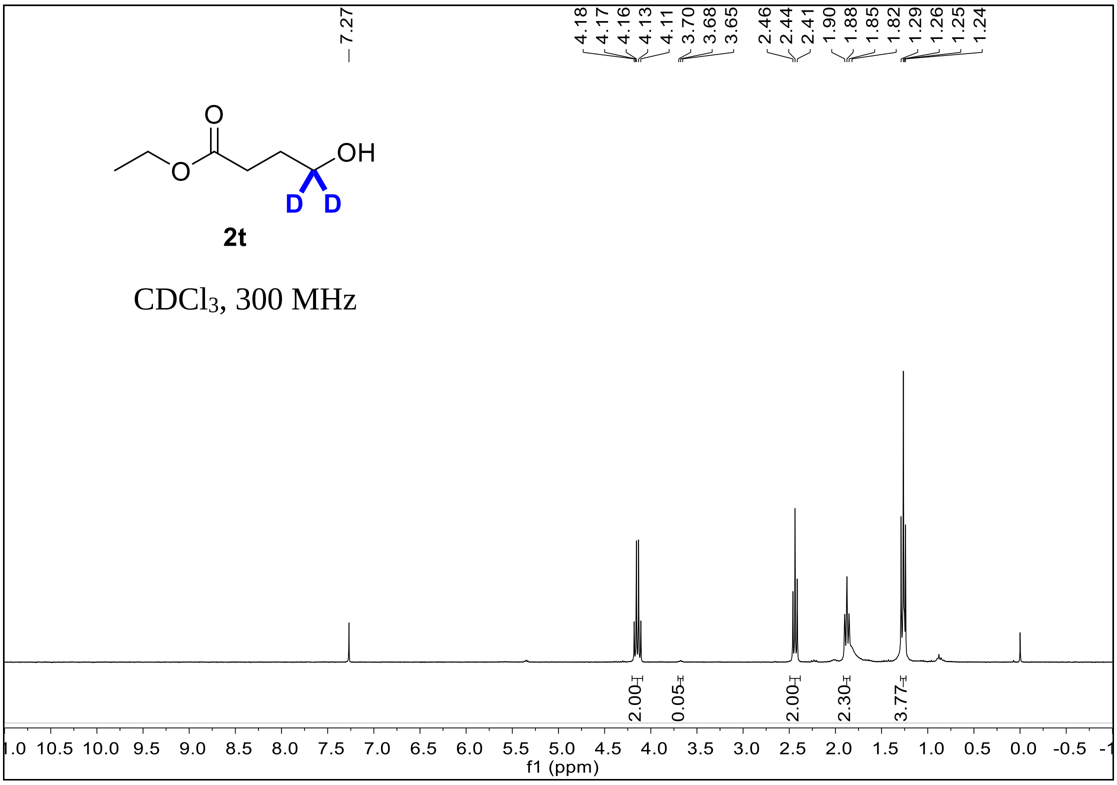
7.27
4.18
4.17
4.16
4.13
4.11
3.70
3.68
3.65
2.46
2.44
2.41
1.90
1.88
1.85
1.82
1.29
1.26
1.25
1.24
O
OH
O
D D
2t
CDCl3, 300 MHz
2.00
0.05
2.00
2.30
3.77
f1 (ppm)

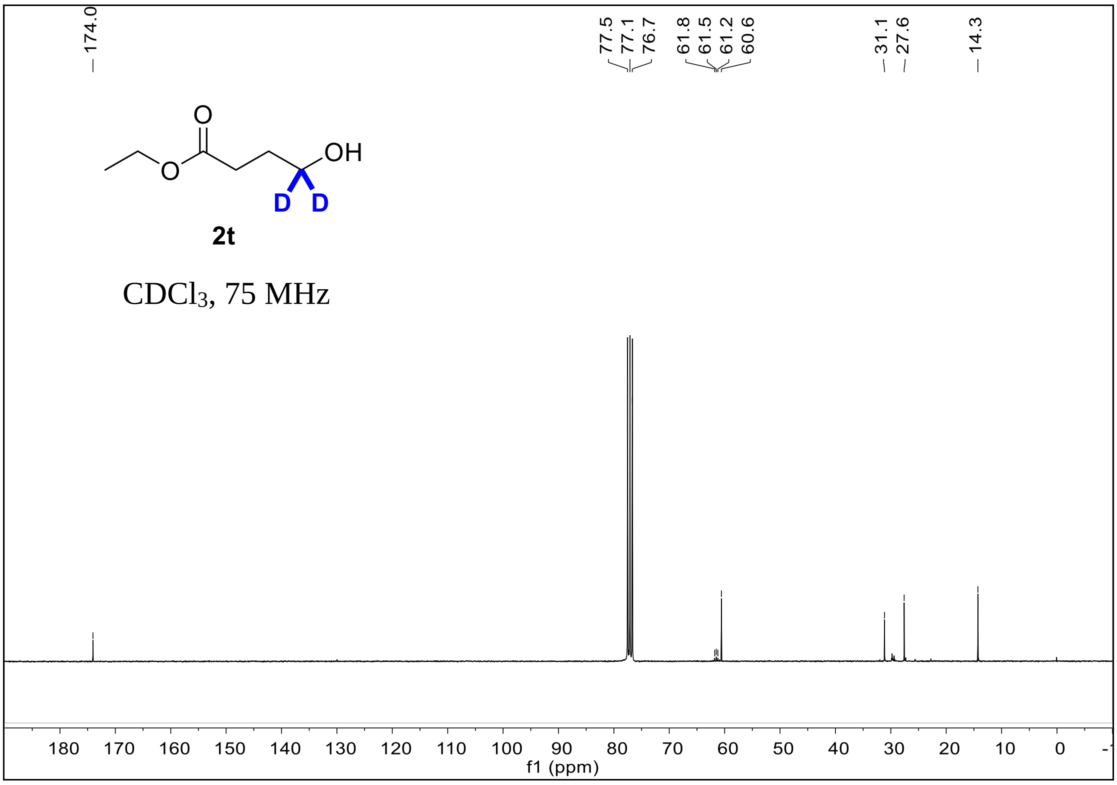
174.0
77.5
77.1
76.7
61.8
61.5
61.2
60.6
31.1
27.6
14.3
O
OH
O
D D
2t
CDCl3, 75 MHz
f1 (ppm)

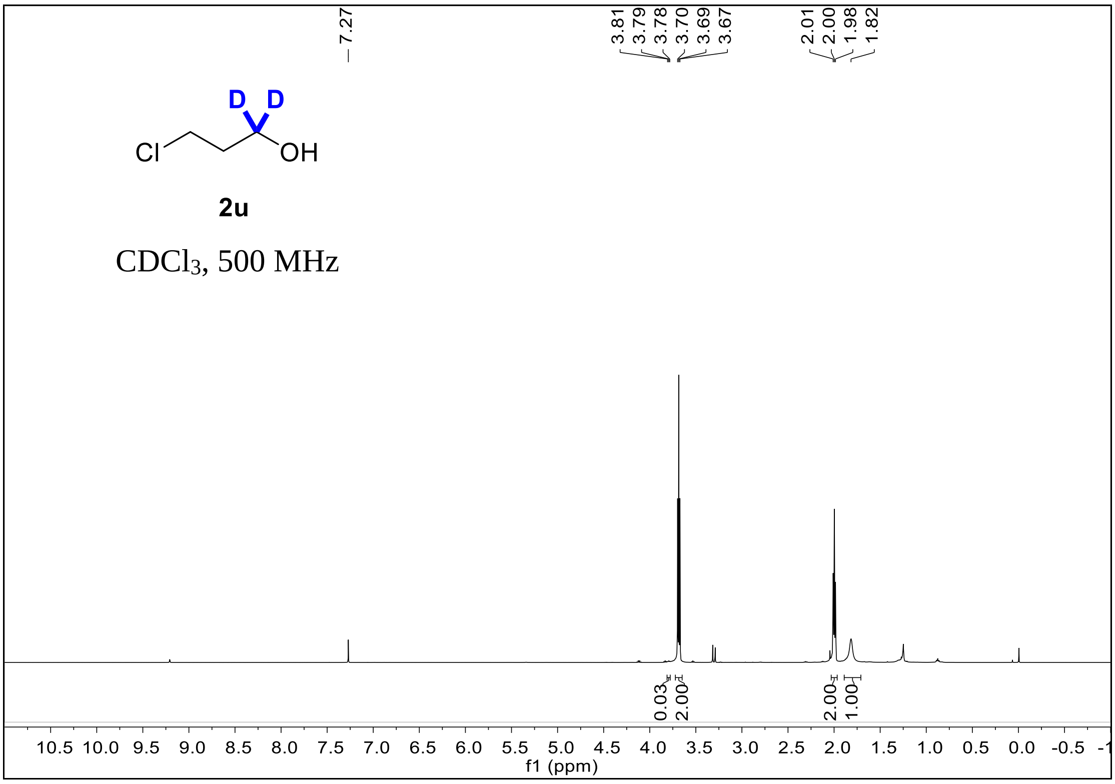
7.27
3.81
3.79
3.78
3.70
3.69
3.67
2.01
2.00
1.98
1.82
D D
Cl
OH
2u
CDCl3, 500 MHz
0.03
2.00
2.00
1.00
10.5 10.0 9.5 9.0 8.5 8.0 7.5 7.0 6.5 6.0 5.5 5.0 4.5 4.0 3.5 3.0 2.5 2.0 1.5 1.0 0.5 0.0 -0.5
f1 (ppm)

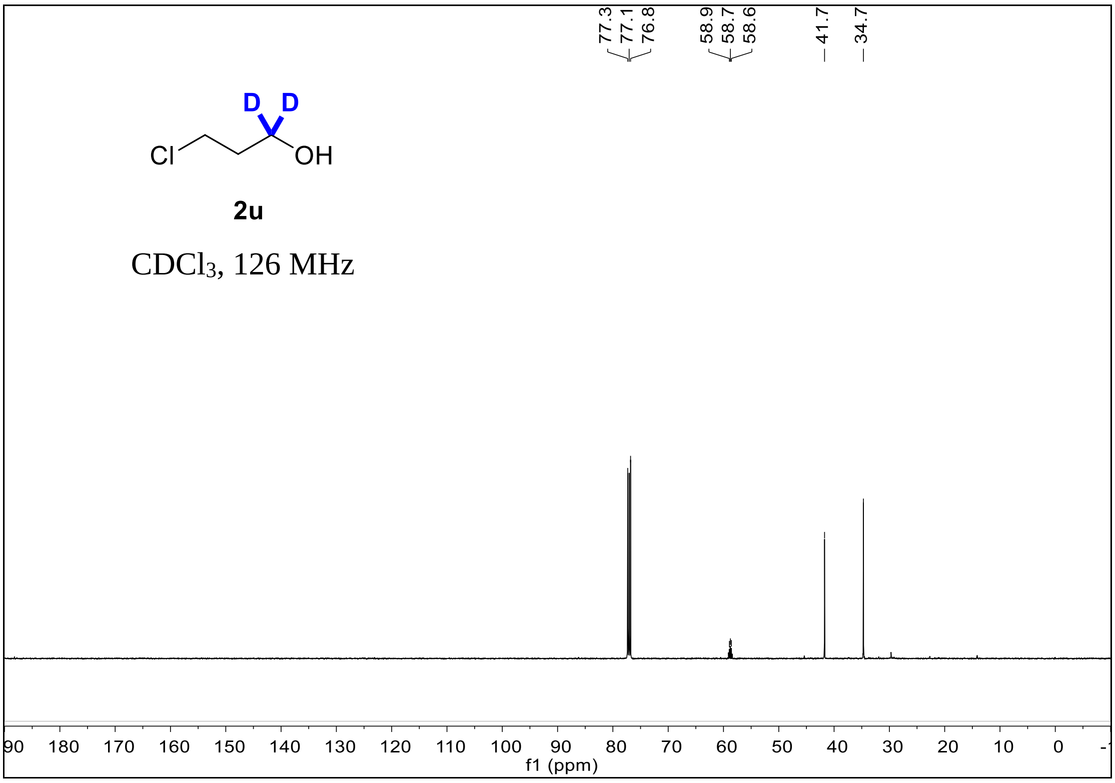
77.3
77.1
76.8
58.9
58.7
58.6
41.7
34.7
D D
Cl
OH
2u
CDCl3, 126 MHz
180 170 160 150 140 130 120 110 100 90 80 70 60 50 40 30 20 10 0
f1 (ppm)

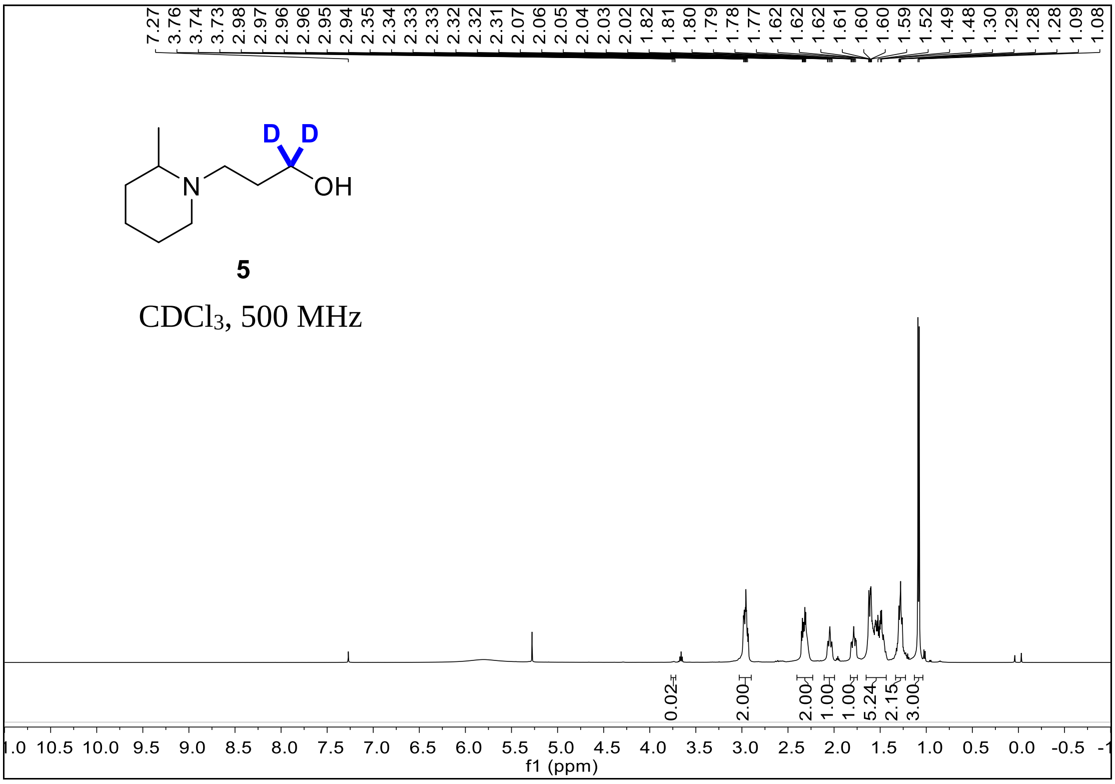
7.27 3.76 3.74 3.73 2.98 2.97 2.96 2.96 2.95 2.94 2.35 2.34 2.33 2.33 2.32 2.32 2.31 2.07 2.06 2.05 2.04 2.03 2.02 1.82 1.81 1.80 1.79 1.78 1.77 1.62 1.62 1.62 1.61 1.60 1.60 1.59 1.52 1.49 1.48 1.30 1.29 1.28 1.28 1.09 1.08
D
D
N
OH
5
CDCl3, 500 MHz
0.02 2.00 2.00 1.00 1.00 5.24 2.15 3.00
f1 (ppm)

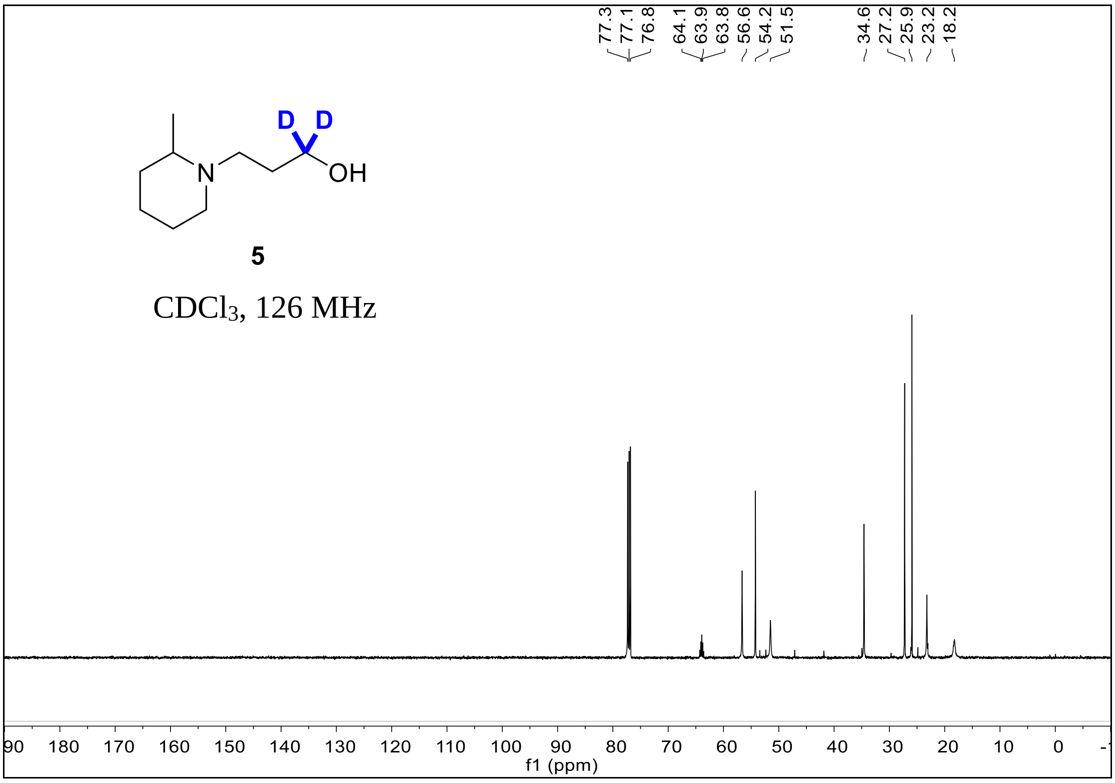
77.3 77.1 76.8 64.1 63.9 63.8 56.6 54.2 51.5 34.6 27.2 25.9 23.2 18.2
D
D
N
OH
5
CDCl3, 126 MHz
f1 (ppm)

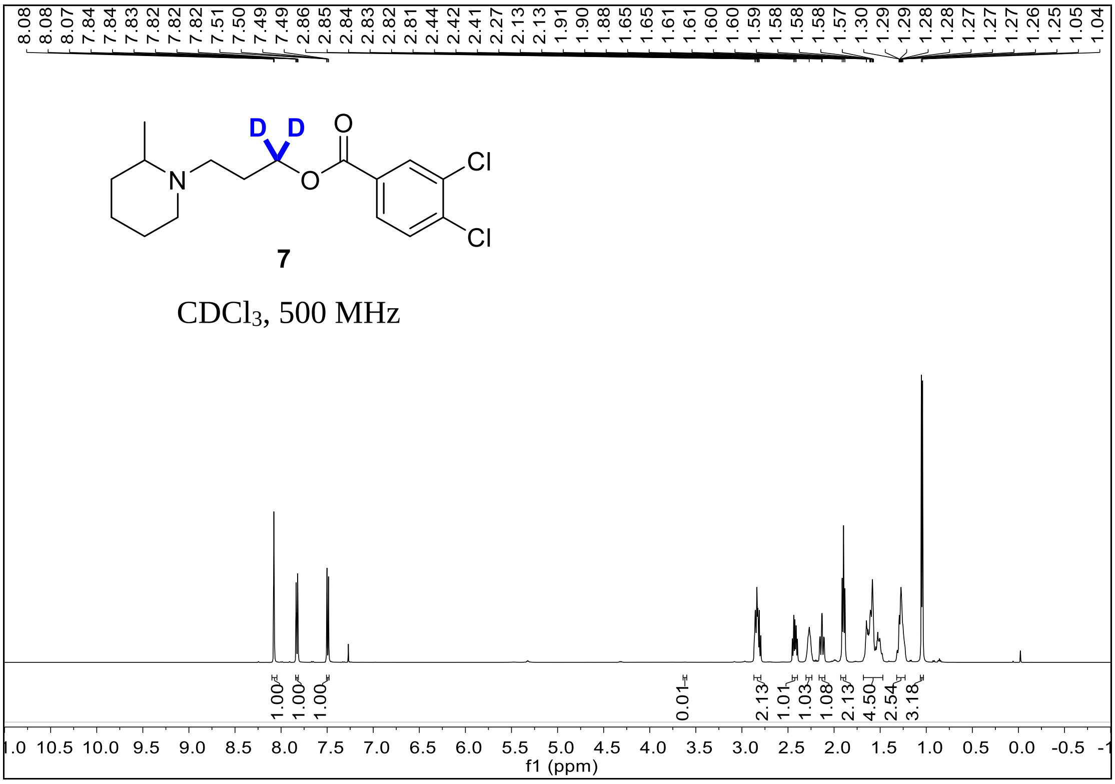
8.08 8.08 8.07 7.84 7.84 7.83 7.82 7.82 7.82 7.51 7.50 7.49 7.49 2.86 2.85 2.84 2.83 2.82 2.81 2.44 2.42 2.41 2.27 2.13 2.13 1.91 1.90 1.88 1.65 1.65 1.61 1.61 1.60 1.60 1.59 1.58 1.58 1.57 1.30 1.29 1.29 1.28 1.28 1.27 1.27 1.27 1.26 1.25 1.05 1.04
D D
O
N
O
Cl
Cl
7
CDCl3, 500 MHz
1.00 1.00 1.00 0.01 2.13 1.01 1.03 1.08 2.13 4.50 2.54 3.18
1.0 10.5 10.0 9.5 9.0 8.5 8.0 7.5 7.0 6.5 6.0 5.5 5.0 4.5 4.0 3.5 3.0 2.5 2.0 1.5 1.0 0.5 0.0 -0.5
f1 (ppm)

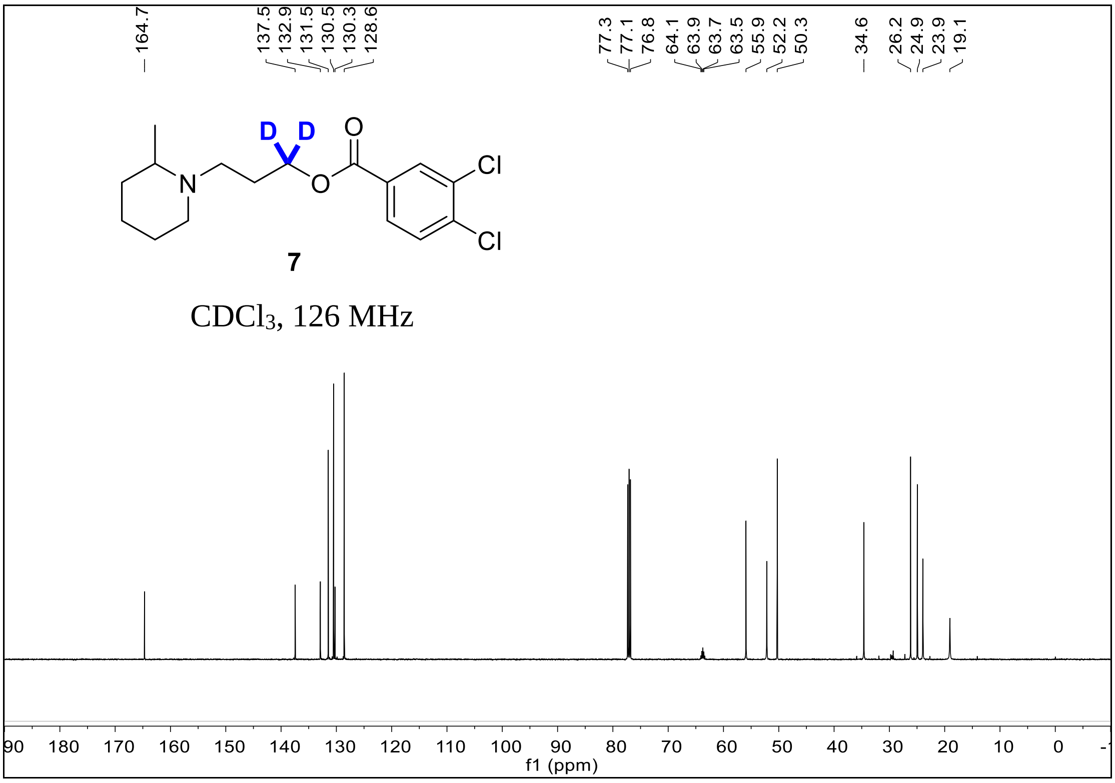
164.7
137.5 132.9 131.5 130.5 130.3 128.6
77.3 77.1 76.8 64.1 63.9 63.7 63.5 55.9 52.2 50.3
34.6 26.2 24.9 23.9 19.1
D D
O
N
O
Cl
Cl
7
CDCl3, 126 MHz
90 180 170 160 150 140 130 120 110 100 90 80 70 60 50 40 30 20 10 0
f1 (ppm)
